# Blockchain-Based Dynamic and Revocable Consent for Secondary Health Data Use: Systematic Review

**DOI:** 10.2196/88536

**Published:** 2026-06-22

**Authors:** Sudip Phuyal, Manila Bhandari, Rabindra Bista, João Carlos Ferreira

**Affiliations:** 1 Information Sciences, Technology and Architecture Research Center (ISTAR), Iscte – Instituto Universitário de Lisboa, Lisbon, Portugal; 2 Department of Computer Science and Engineering, Kathmandu University, Dhulikhel, Nepal; 3 Faculty of Logistics, Molde University College, Britvegen 2, Molde, 6410, Norway, 47 71214000; 4 Inov Inesc, Lisbon, Portugal

**Keywords:** blockchain, dynamic consent, revocable consent, health care data sharing, data privacy, auditability, General Data Protection Regulation, GDPR, European Health Data Space, EHDS

## Abstract

**Background:**

The secondary use of health data holds substantial potential for advancing biomedical research, strengthening population health analytics, and enabling artificial intelligence–driven decision-making support. Yet, ensuring that such reuse respects patient autonomy, privacy, and regulatory obligations remains a major challenge. Conventional consent mechanisms are typically static, difficult to revoke, and offer limited transparency or accountability after data disclosure.

**Objective:**

This review aimed to systematically examine blockchain-based frameworks that enable dynamic, auditable, and revocable consent for the secondary use of health data.

**Methods:**

A structured literature search was conducted in PubMed, Scopus, and Web of Science covering the period 2020 to 2025. Following PRISMA (Preferred Reporting Items for Systematic Reviews and Meta-Analyses) guidelines, 55 peer-reviewed studies meeting predefined inclusion criteria were analyzed. Data extraction focused on four dimensions: (1) consent life cycle management, (2) auditability and traceability, (3) usability and patient empowerment, and (4) legal and ethical alignment.

**Results:**

Findings indicate that blockchain technologies provide a robust foundation for automating consent life cycles, ensuring immutable auditability, and enabling decentralized patient control. Most frameworks used smart contracts, decentralized identifiers, and verifiable credentials to implement programmable and verifiable consent processes. Nevertheless, key challenges persist, including limited usability testing, complexities in real-time revocation propagation, interoperability gaps with clinical systems, and tensions with regulatory requirements such as the General Data Protection Regulation right to erasure. Only a small subset of studies reported real-world deployments or user-centered evaluations.

**Conclusions:**

Blockchain offers substantial promise for improving the trustworthiness, transparency, and accountability of consent management for secondary health data use. However, wider adoption requires human-centered design approaches, stronger interoperability through standards such as Fast Healthcare Interoperability Resources, verifiable credentials, and consent receipts, and clearer legal guidance for compliance. Future research should prioritize integrating blockchain-enabled consent infrastructures into national and cross-border digital health ecosystems such as the European Health Data Space to support secure, patient-controlled, and ethically governed secondary data use.

## Introduction

### Background

The digitalization of health care has expanded how patient data are used and shared, raising important questions about consent, privacy, and individual control. While large-scale reuse of health data enables advances in biomedical research, population health analytics, and data-driven innovation, traditional consent models often fall short in this evolving landscape [1,2]. Conventional approaches are typically static, difficult to revoke, and provide limited transparency once data have been shared, making them poorly suited for continuous, cross-institutional, and cross-border secondary use of health data.

The digitalization of health care has expanded how patient data are used and shared, raising important questions about consent, privacy, and individual control. While large-scale reuse of health data enables advances in biomedical research, population health analytics, and data-driven innovation, traditional consent models often fall short in this evolving landscape [1,2]. Conventional approaches are typically static, difficult to revoke, and provide limited transparency once data have been shared, making them poorly suited for continuous, cross-institutional, and cross-border secondary use of health data.

Recent regulatory developments, notably the European Health Data Space (EHDS) Regulation, further reinforce the need for interoperable and trustworthy consent infrastructures for secondary use of health data. Acting as lex specialis over the General Data Protection Regulation (GDPR) in this context, the EHDS introduces dedicated governance mechanisms [3], access frameworks, and infrastructure requirements for health data reuse within the European Union. While the GDPR establishes baseline principles for lawful processing, including informed and revocable consent, the EHDS places additional emphasis on harmonized procedures, institutional accountability, and cross-border operability [4].

Recent implementation guidance further clarifies how patient rights under the EHDS should be operationalized in practice. In particular, the second joint action Towards the European Health Data Space (TEHDAS 2) guideline for Health Data Access Bodies specifies common principles and procedures for implementing opt-out mechanisms for the secondary use of electronic health data, emphasizing transparency, harmonized national processes, and citizen-facing tools to exercise opt-out rights [3]. This guidance highlights that EHDS compliance depends not only on legal provisions but also on concrete organizational and technical processes capable of managing consent and opt-out at scale.

At the same time, emerging technologies such as blockchain and decentralized identity offer new architectural possibilities for enforcing consent policies, maintaining immutable audit trails, and enabling patient-centric control without reliance on central intermediaries [5-7]. Blockchain-based systems can provide tamper-evident records, shared governance across institutions, and programmable enforcement of consent conditions through smart contracts, while decentralized identity mechanisms allow individuals to retain control over their identifiers and consent credentials.

Several recent surveys have explored blockchain in health care or digital consent in isolation; however, they typically address broad application domains or focus on usability, ethical, or governance perspectives without systematically analyzing how blockchain-based architectures support dynamic and revocable consent specifically for the secondary use of health data. As a result, there remains a fragmented understanding of how consent life cycles are technically implemented, how revocation is enforced across distributed systems, and how such frameworks align with evolving regulatory requirements under the GDPR and the EHDS [6-10]. This gap motivates this systematic review.

Unlike prior reviews that examine blockchain in health care broadly or address digital consent primarily from ethical or governance perspectives, this review analyzes dynamic and revocable consent as an enforceable life cycle across distributed systems, integrating architectural, operational, usability, and regulatory dimensions.

Accordingly, the aim of this systematic review was to examine and synthesize blockchain-based frameworks that support dynamic, auditable, and revocable consent for the secondary use of health data. Specifically, this review analyzes how existing approaches implement consent life cycle management, enable auditability and traceability, support patient empowerment and usability, and align with legal and ethical requirements under the GDPR and the emerging EHDS. By consolidating evidence from peer-reviewed studies published between 2020 and 2025, this review seeks to identify prevailing architectural patterns, implementation gaps, and priority directions for future research and real-world deployment.

This paper is organized as follows. The *Methods* section describes the systematic review protocol, including the search strategy, eligibility criteria, and data extraction and synthesis procedures. The *Results* section presents the findings of the review, organized around key analytical dimensions related to consent life cycle management, auditability, usability, and legal-ethical alignment. The *Discussion* interprets these findings in relation to existing literature, regulatory frameworks, and practical implications for the secondary use of health data.

### Background and Rationale

The rapid advancements in digital technology over the past three decades have led to the emergence of digital health, which is revolutionizing health care [11]. The digital transformation of health care has redefined how clinical and research data are collected, managed, and reused. With electronic health records (EHRs) and other digital health systems being adopted so quickly, health care data have grown exponentially [12]. The EHRs, biobanks, and data-driven analytics platforms now form the backbone of modern health care systems by supporting clinical decision-making, personalized medicine, and population-level insights. Global policy initiatives, such as the World Health Organization’s Global Strategy on Digital Health [2], emphasize the need for secure and interoperable infrastructures that enable the ethical data exchange and innovation.

Alongside this advancement, the secondary use of health data beyond direct patient treatment has become a key component of biomedical research, artificial intelligence (AI) model development, and public health surveillance [1]. However, these benefits raise the fundamental questions about patient autonomy, privacy, and consent governance. Legal frameworks such as the GDPR require that personal data be handled transparently. They also specify that consent must always be specific, informed, and easy to withdraw [13]. In the European Union, the recently adopted EHDS Regulation further establishes a dedicated legal framework for the secondary use of health data, acting as lex specialis over the GDPR in this domain and introducing cross-border governance and access mechanisms [4].

Despite these regulatory advances, current consent management models remain largely static [14]. Usually, the individuals sign a one-time authorization form that gives them data controllers general authorization to reuse their data, but they are not given tools to control or withdraw their consent in a more precise way [15]. The centralized databases used for storing consent records often face a lack of transparency and auditability. In addition, they struggle to maintain real-time synchronization across the institutions, which creates single points of failure and reduces overall accountability. These weaknesses highlight the need for systems that support continuous, verifiable, and user-driven consent throughout the data life cycle.

From a technical perspective, these requirements challenge traditional centralized architectures and motivate the exploration of distributed trust infrastructures that can provide tamper-evident records, shared governance, and automated enforcement of consent policies across organizational boundaries.

In this context, blockchain and distributed ledger technologies (DLTs) have emerged as promising enablers due to their inherent properties of immutability, decentralization, and programmable logic. However, the choice of blockchain deployment model is critical in health care settings. Public, permissionless blockchains offer high transparency but raise concerns regarding scalability, governance, and the exposure of even pseudonymized metadata on global infrastructures, which may conflict with data minimization and purpose limitation principles.

Consequently, most health care–oriented frameworks favor permissioned or consortium DLTs, where participating nodes are operated by trusted health care authorities or institutions and where fine-grained access control and governance mechanisms can be enforced. Such models better align with regulatory expectations under GDPR and EHDS, while still enabling shared auditability and decentralized trust. These considerations underscore the need for consent management solutions that are not only legally compliant but also technically capable of operating in distributed, cross-institutional health care ecosystems. This background motivates the investigation of blockchain-based frameworks as potential trust layers for enabling dynamic, auditable, and revocable consent in the secondary use of health data.

### Blockchain Deployment Models and Regulatory Constraints

Although blockchain technologies are frequently proposed as trust infrastructures for health care consent management, the choice of deployment model is critical. Public, permissionless blockchains, while offering high transparency and openness, are generally unsuitable for managing consent-related metadata in health care settings. Even when only pseudonymized identifiers, public keys, or decentralized identifiers (DIDs) are stored on-chain, the global replication of such metadata across open infrastructures lacks a clear need-to-know justification and may conflict with the GDPR principles of data minimization and purpose limitation.

Consequently, most health care–oriented consent management frameworks adopt permissioned or consortium-based DLTs, where participating nodes are operated by trusted health care institutions, public authorities, or regulated intermediaries. These models enable fine-grained access control, governance, and institutional accountability while still preserving shared auditability and tamper-evident records across organizational boundaries. Such architectural choices align more closely with GDPR requirements and with the governance expectations introduced by the EHDS framework for the secondary use of health data.

### Evolution of Consent and the Need for Dynamic Models

Traditional consent models were designed for one-time clinical visits or specific research studies [15,16]. In an era of continuous data flows and distributed analytics, these static approaches have become increasingly inadequate. Individuals rarely could modify or revoke consent as data are repurposed for new studies, shared across borders, or integrated into AI training pipelines [17]. This gap between what patients expect and what technology currently delivers can weaken trust in the digital health ecosystems.

To address these concerns, dynamic consent was introduced as both an ethical and technical solution that allows individuals to modify, refine, or revoke their data sharing choices at any time through the user-friendly interactive platforms [18]. By enabling ongoing engagement and communication between data subjects and controllers, dynamic consent promotes transparency, autonomy, and contextual decision-making. It also aligns with key principles of the GDPR, particularly Article 7, which ensures revocability, and Article 17, which gives individuals the right to have their data deleted. Within the European context, these principles are further operationalized for secondary use through the EHDS Regulation, which emphasizes patient rights, governance, and cross-border data reuse under harmonized access mechanisms.

However, unlike primary use where consent is often the main legal basis, the EHDS introduces a governance model for secondary use in which data access may rely on specific legal bases but remains subject to a citizen’s right to opt out. The TEHDAS guideline elaborates how Health Data Access Bodies should implement such opt-out mechanisms in practice, including registries and user interfaces for objections. This distinction is crucial for blockchain-based dynamic consent systems, which must interoperate with opt-out governance rather than assume that consent alone governs secondary use [3].

In contrast, most dynamic-consent implementations rely on centralized architectures that provide limited verifiability and poor interoperability across institutions. These systems may log user actions but cannot guarantee immutability, traceability, or real-time enforcement of revocation [19]. As secondary use increasingly involves multi-institutional and cross-border data flows, such limitations pose not only technical but also governance and compliance challenges. In particular, the lack of automated propagation of consent changes across distributed data custodians undermines the practical realization of revocability required by both GDPR and EHDS.

As secondary data use expands across jurisdictions and organizations, these limitations pose both technical and ethical challenges. This evolution highlights the need for consent infrastructures that can (1) maintain a shared and tamper-evident history of consent decisions, (2) support fine-grained and time-bound authorizations, and (3) enable verifiable enforcement of revocation across organizational boundaries.

These requirements motivate the exploration of blockchain-based approaches as potential enablers of dynamic, auditable, and decentralized consent management in health care.

### Blockchain Foundations for Dynamic Consent

Blockchain offers architectural features that can address many of the limitations of centralized consent management [5]. Consent events, such as granting, updating, and revoking permissions, can be recorded in a tamper-proof manner verifiable by all the participants’ nodes, which is immutability, transparency, and decentralization. These properties enable shared trust across organizations without relying on a single central authority.

Smart contracts are self-executing programs that are stored on the blockchain that can automatically manage consent by granting or withdrawing access based on predefined conditions, such as specific time frames or user decisions [20]. This makes it possible to handle consent in real time, reducing the need for third-party involvement and manual reconciliation between data controllers.

Other technologies, such as DIDs and verifiable credentials (VCs), further enhance this process by enabling individuals to control their own digital identities [21]. With DIDs, users can create and manage their own digital identities without depending on the centralized systems. Meanwhile, VCs also make it possible to issue and share digitally signed proofs, such as consent records, which others can verify without revealing unnecessary personal information. Together, these tools enable a privacy-preserving, patient-centric consent framework where individuals maintain verifiable control over their data without exposing personal identifiers on-chain.

In most cases, blockchain is used as a coordination and auditing layer rather than a data storage layer. The sensitive health information remains off-chain, while only hashed references, metadata, and consent proofs are recorded on the ledger. This hybrid design supports GDPR’s principles, such as data minimization and purpose limitation, and is also aligned with EHDS expectations for interoperable and trustworthy secondary-use infrastructures.

Accordingly, hybrid on-chain or off-chain architectures represent a baseline design pattern across health care blockchain systems, adopted from early proposals to recent implementations to address scalability and privacy constraints.

Although these technologies show potential for creating secure, adaptable, and revocable consent systems, it remains unclear how well they perform in real health care environments. Challenges persist in synchronizing on-chain consent states with off-chain policy enforcement points (PEPs), ensuring timely revocation propagation, and integrating with existing health information systems. This highlights the need for a comprehensive review of current research to examine how the blockchain is being applied to dynamic consent and to consider the technical, ethical, and legal issues that accompany it.

### Identified Research Gaps

Blockchain and consent management have each been widely studied within digital health research. However, their intersection, particularly with respect to dynamic and revocable consent for the secondary use of health data, remains insufficiently explored. Most blockchain-focused studies adopt a broad perspective, emphasizing applications such as EHR management, supply chain traceability, or digital identity, while treating consent as a static or peripheral feature. These approaches rarely conceptualize consent as a programmable life cycle, enabling patients to grant, monitor, modify, and revoke permissions over time.

In contrast, research on dynamic consent has predominantly emerged from ethical, legal, and user experience perspectives, emphasizing patient autonomy, transparency, and trust. While this body of work provides important insights into participatory consent models, it often lacks a detailed examination of the technical infrastructures required to operationalize such models at scale. Key aspects, including verifiable audit trails, enforceable revocation, interoperability across heterogeneous data custodians, and tamper-resistant governance mechanisms, are seldom addressed in depth.

Across the primary literature, blockchain-based proposals for consent management are highly heterogeneous, spanning domains such as clinical trials, personal health records (PHRs), genomics, and health information exchange. Despite this diversity, there is a notable lack of systematic synthesis focused specifically on secondary-use governance, where consent must be enforceable across institutional and jurisdictional boundaries and over extended time horizons. As a result, the design patterns, architectural trade-offs, and operational limitations of blockchain-enabled dynamic consent systems for secondary health data reuse remain poorly consolidated.

More recent studies have proposed frameworks combining blockchain with decentralized identity technologies, including DIDs and VCs. However, these approaches vary considerably in architectural design, regulatory assumptions, and levels of empirical validation. Few studies move beyond proof-of-concept implementations to evaluate performance in realistic cross-institutional or cross-border settings. In particular, automated revocation propagation, latency guarantees, coupling with off-chain PEPs, and scalability under operational workloads remain weakly demonstrated.

Finally, although alignment with the GDPR is frequently asserted, the implications of the EHDS Regulation as lex specialis for secondary use introducing dedicated governance bodies, access authorization mechanisms, and cross-border infrastructures are only sporadically addressed. This creates uncertainty regarding how blockchain-based consent models can integrate with emerging European data space architectures.

Accordingly, this study addresses these gaps by providing a PRISMA (Preferred Reporting Items for Systematic Reviews and Meta-Analyses)–based systematic review of literature published between 2020 and 2025, applying a structured analytical framework and quality assessment to evaluate blockchain-based dynamic consent architectures for secondary use of health data. By focusing on consent life cycle management, auditability, usability evidence, and legal-ethical alignment, this review consolidates fragmented evidence and identifies the technical, evaluative, and governance limitations constraining the operational maturity of existing approaches.

### Related Work and Critical Comparison With Existing Reviews

Several recent reviews and surveys have examined blockchain and consent in health care from complementary but partial perspectives. However, none provide a unified analysis of dynamic and revocable consent life cycles for secondary health data use, grounded simultaneously in blockchain-based enforcement mechanisms and emerging European regulatory requirements.

For instance, Hang et al [6] reviewed blockchain applications in clinical trials, emphasizing transparency and traceability, but treated consent primarily as a supporting mechanism rather than as a programmable life cycle with explicit revocation semantics. Similarly, Baysal et al [7] and other broad surveys map blockchain-based health care systems across diverse use cases, yet do not isolate consent management for secondary use as a distinct analytical focus, nor do they assess the enforceability or governance implications in depth.

From the consent-oriented literature, Schmidt et al [8] presented a scoping review of informed consent in digital health, identifying challenges related to consent acquisition and management. However, this work does not engage with blockchain architectures, smart contracts, or decentralized enforcement mechanisms. Likewise, Kassam et al [9] and Cumyn et al [10] synthesized patient and public perspectives on digital consent and transparency in secondary data use, offering valuable socioethical insights but without examining technical design choices or system-level trade-offs.

Other recent surveys, including Phuyal et al [1], provide comprehensive overviews of blockchain in health care but do not analyze consent life cycles, revocation propagation, or patient-controlled secondary use governance. Across these reviews, regulatory considerations are typically limited to high-level discussions of GDPR compliance, with minimal treatment of the EHDS Regulation and its implications for secondary use authorization, governance bodies, and cross-border interoperability.

To situate this review within this landscape, Table 1 provides a structured comparison of recent review and survey papers intersecting blockchain, digital consent, and secondary use of health data. As summarized, existing reviews either focus broadly on blockchain applications without analyzing consent life cycle enforceability or examine digital consent without addressing blockchain-based technical enforcement and secondary use governance. In contrast, this review uniquely integrates (1) smart contract–based consent life cycle management, (2) auditability and traceability mechanisms, (3) usability and empirical evaluation evidence, and (4) legal alignment with both GDPR and the EHDS Regulation, supported by a PRISMA methodology and formal quality assessment across 55 studies.

This comparative analysis demonstrates that this review does not duplicate prior surveys but instead provides an integrative assessment of consent life cycle enforceability and governance readiness for secondary health data use, which has not been systematically examined in earlier secondary studies.

Taken together, prior reviews reflect the gradual evolution of consent research from static, paper-based authorization toward digital and dynamic consent models and more recently toward decentralized and blockchain-enabled infrastructures. Early work on dynamic consent primarily emphasized ethical engagement, communication, and patient trust, while blockchain-focused health care surveys concentrated on data integrity, traceability, and access control. However, these strands have largely developed in parallel. As a result, the technical enforceability of dynamic and revocable consent across distributed systems, particularly for secondary use of health data, has remained insufficiently synthesized in the literature.

**Table 1.** Comparison of this review with recent related surveys.

Author, year	Scope	Blockchain focus	Dynamic or revocable consent	Secondary use focus	EHDS^a^ or GDPR^b^ analysis	Usability and empirical evaluation	Quality assessment	This review’s added value
Hang et al (2022) [6]	Blockchain in clinical trials	Yes	Yes	Focused on trials	GDPR only	No	No	Lacks life cycle and revocation analysis
Baysal et al (2023) [7]	Multivocal blockchain in health care	Yes	Yes	Broad	No	No	Yes	Broad mapping, no consent depth
Schmidt et al (2025) [8]	Digital health consent (scoping)	Partial	Yes	Yes	GDPR only	No	No	No blockchain architecture analysis
Kassam et al (2023) [9]	Patient perspectives on digital consent	No	Yes	Yes	No	Yes	No	Human factors only
Cumyn et al (2023) [10]	Transparency in secondary use	No	Partially	Yes	Yes	Yes	No	Governance without technology
Phuyal et al (2025) [1]	Blockchain in health care (survey)	Yes	No	No	Yes	No	No	No consent life cycle or revocation
This review	Blockchain+dynamic consent for secondary use	Yes	Yes	Yes	Yes	Yes	Yes	End-to-end life cycle, revocation propagation, auditability, EHDS focus

a EHDS: European Health Data Space.

b GDPR: General Data Protection Regulation.

### Scope and Contribution of the Review

The purpose of this review is to deliver a thorough and systematic examination of blockchain-based frameworks that support dynamic, auditable, and revocable consent for the secondary use of health data. It adds to current studies by investigating how these systems manage the full life cycle of consent in health care using blockchain and decentralized identity technologies. It also evaluates the system’s technical reliability, usability, and compliance with regulatory standards. In bringing these findings together, this review highlights common design approaches and enduring challenges across these separate studies. By integrating findings from technological, ethical, and governance perspectives, it explains how blockchain can enable patient-centric consent systems. It also makes practical recommendations for researchers, system developers, health care providers, and policymakers.

More specifically, this review makes 4 key contributions. First, it provides a PRISMA 2020–compliant synthesis of recent literature published between 2020 and 2025, focusing explicitly on blockchain-enabled dynamic and revocable consent for the secondary use of health data. Second, it applies a multidimensional analytical framework, encompassing consent life cycle management, auditability, usability, and legal-ethical alignment, complemented by a structured quality assessment of the included studies. Third, it identifies recurring architectural patterns such as hybrid on-chain or off-chain designs and the use of DIDs and VCs and analyzes their implications for interoperability, revocation enforcement, and compliance with GDPR and the emerging EHDS framework. Finally, it highlights critical gaps and future research directions required to advance these systems from conceptual prototypes toward operational health care infrastructures.

### Objectives and Aims of the Review

The aim of this systematic review was to synthesize and critically evaluate blockchain-based frameworks that support dynamic, auditable, and revocable consent for the secondary use of health data. Specifically, the review seeks to (1) characterize the architectural and operational approaches used to model and enforce consent life cycles; (2) assess how auditability, provenance, and accountability are implemented across distributed systems; and (3) evaluate the extent to which proposed solutions address usability, interoperability, and regulatory alignment, including compliance with the GDPR and the emerging EHDS.

Together, these objectives frame the review’s analytical focus on both technical maturity and practical readiness for real-world deployment.

## Methods

### Overview

This systematic review was conducted to identify, analyze, and synthesize peer-reviewed literature on blockchain-based frameworks that enable dynamic, auditable, and revocable consent for the secondary use of health data. The review followed the PRISMA guidelines (Checklist 1) to ensure transparency and reproducibility. The methodological steps included defining eligibility criteria, developing a comprehensive search strategy, screening and selecting studies, extracting and synthesizing data, performing a structured quality assessment, and classifying findings according to a conceptual analytical framework.

### Review Design

A systematic literature review approach was selected to provide a comprehensive and structured overview of the current research landscape. This design supports evidence-based synthesis by integrating findings from diverse studies that address similar research questions across technical, ethical, and regulatory dimensions. The review focused on primary research papers, system proposals, prototypes, and evaluations that describe blockchain-based consent management models relevant to health care data reuse. Conceptual papers were included only if they contributed a defined architectural or governance model. The review protocol was defined in advance to minimize selection bias and ensure consistency across screening, data extraction, and synthesis phases.

### Eligibility Criteria

Eligibility criteria were established using the PICOS (population, intervention, comparison, outcome, and study type) framework, which ensures a consistent and unbiased selection process.

Inclusion criteria were as follows:

- Studies proposing or evaluating blockchain or distributed ledger–based consent systems within health care contexts
- Systems explicitly supporting dynamic consent, including granular, time-bound, or revocable authorization
- Studies addressing secondary use of health data, such as in research, AI model development, clinical trials, or data analytics
- Peer-reviewed journal publications between January 2020 and October 2025
- Publications written in English

To improve clarity and reproducibility, the inclusion criteria were operationalized as follows:

- Population: health care stakeholders and patient data contexts
- Intervention: blockchain or DLT-based consent management mechanisms
- Comparison: not mandatory, as most studies proposed novel frameworks
- Outcomes: support for dynamic, auditable, and revocable consent and/or reported technical or usability evaluation and
- Study type: peer-reviewed empirical, prototype, or architectural studies

Likewise, the research was set with the following exclusion criteria to filter out the irrelevant articles:

- Studies unrelated to health care or consent management
- Solutions limited to static, one-time consent without revocation or modification features
- Opinion pieces, editorials, or commentaries without technical or empirical contribution
- Duplicates or non–peer-reviewed materials such as white papers and theses or dissertations
- Conference papers were excluded unless extended journal versions were available to ensure sufficient methodological detail and maturity of the included studies.

### Information Sources and Search Strategy

The literature search was performed across 3 major electronic databases known for health informatics and blockchain research: PubMed, Scopus, and Web of Science. A Boolean search string was developed and adapted to each database’s syntax, combining key terms related to consent management, blockchain, and health data reuse. The core search expression used was as follows:

 (“dynamic consent” OR “revocable consent” OR “granular consent”) AND

 (“blockchain” OR “distributed ledger” OR “DLT”) AND

 (“health” OR “healthcare” OR “medical data”) AND

 (“secondary use” OR “research” OR “data sharing” OR “AI training”)

The core Boolean search strategy was adapted to the syntax and indexing mechanisms of each database. In PubMed, the search was applied to title and abstract fields and supplemented with relevant Medical Subject Headings (MeSH) where available. In Scopus and Web of Science, the search was executed using title, abstract, and keyword fields, with database-specific field tags and operators applied to preserve semantic equivalence across platforms. Truncation and phrase matching were used where supported. These adaptations ensured comprehensive retrieval while maintaining consistency in search intent across databases. The complete database-specific query formulations are available from the authors upon request to support reproducibility.

The final searches were conducted in October 2025, and the results were limited to publications between January 2020 and October 2025. Search fields included titles, abstracts, and keywords where supported by the databases.

Reference lists of included studies were manually screened to identify additional relevant publications not captured by database queries. All records retrieved from the search were exported to a Zotero citation manager [22] for deduplication and structured screening. The complete search strategy and database-specific query adaptations are available upon request to support reproducibility.

### Study Selection Process

The selection process was conducted in 2 main stages:

- Title and abstract screening: Each record was independently reviewed to assess relevance against inclusion criteria. Articles clearly outside the health care or blockchain domain were excluded at this stage.
- Full-text review: Potentially relevant studies were examined in full to confirm eligibility and to ensure that they met the review’s focus on dynamic and revocable consent mechanisms.

Any discrepancies in inclusion decisions were resolved by discussion among authors to reach consensus. The entire process was documented using a PRISMA 2020 flow diagram, which summarizes the number of records identified, screened, excluded, and included in the final analysis.

Specifically, 2 authors (SP and MB) independently screened all titles and abstracts, and disagreements were resolved through discussion, consulting with the authors RB and JCF when consensus could not be reached. The same procedure was applied during full-text assessment.

Interrater agreement between the 2 reviewers was assessed to evaluate screening consistency. Agreement was substantial, with a Cohen κ coefficient of 0.78 during title and abstract screening and κ of 0.81 during the full-text eligibility assessment, indicating a high level of concordance. Discrepancies were resolved through discussion and, where necessary, consultation with senior authors to reach consensus.

### Data Extraction and Coding

A standardized data extraction form was designed to ensure consistency in collecting relevant details from each study. The following key attributes were extracted:

- Bibliographic details: authors, publication year, source, and country of origin
- Use case: health care domain, population, and purpose of data sharing
- Blockchain architecture: platform, consensus model, and smart contract use
- Identity management: use of DIDs, VCs, or public key infrastructure
- Consent features: life cycle stages supported (grant, modify, and revoke), granularity, and enforcement mechanisms
- Auditability mechanisms: on-chain logging, event tracking, hash anchoring, or hybrid verification models
- Usability aspects: interface design, patient engagement, and evaluation methods
- Compliance and ethics: references to GDPR, EHDS, or ethical principles
- Reported challenges: technical, organizational, or legal barriers to implementation

Additional fields were included to capture blockchain deployment models (public, permissioned, or consortium), hybrid on-chain or off-chain designs, and any mechanisms for automated revocation propagation or integration with clinical systems (eg, HL7 [Health Level 7] FHIR [Fast Healthcare Interoperability Resources]).

Extracted data were entered into a structured spreadsheet and cross-verified for accuracy and completeness. Data extraction was performed independently by 2 reviewers on a subset of studies to validate consistency, with disagreements resolved through discussion.

### Analytical Framework and Data Synthesis

The synthesis process combined quantitative description with qualitative thematic analysis to identify design patterns and conceptual trends across studies. The analysis was guided by four key conceptual dimensions derived from the literature and regulatory frameworks:

1. Consent life cycle management: how systems model, enforce, and update consent states, including mechanisms for revocation
2. Auditability and traceability: the extent to which systems provide immutable and verifiable records of consent and data access
3. Usability and patient empowerment: the user-facing design and degree of patient control supported by interfaces or wallets
4. Legal and ethical alignment: compliance with data protection laws and ethical requirements related to transparency, purpose limitation, and revocability

Studies were coded against these dimensions and grouped into thematic clusters to highlight common technical approaches, gaps, and best practices. Where relevant, architectural diagrams and comparative tables were used to illustrate design similarities and differences.

Quantitative findings, such as the prevalence of blockchain platforms or identity mechanisms, were summarized using descriptive statistics, while qualitative findings were narratively synthesized to contextualize patterns and highlight challenges. The combined synthesis enabled cross-dimensional analysis, for example, linking architectural choices to observed usability or compliance limitations.

### Quality Assessment

To ensure methodological rigor, each included study was evaluated for quality using a set of predefined criteria adapted from digital health and software engineering review frameworks. These criteria assessed the following:

- Clarity of objectives and problem definition
- Transparency in describing the system’s architecture or implementation
- Explicit discussion of consent mechanisms and revocation of support
- Alignment with privacy or regulatory standards
- Evidence of validation, testing, or pilot evaluation

Each criterion was scored as 0 (not met), 0.5 (partially met), or 1 (fully met). Scores were summed and normalized to a 0 to 1 scale to derive an overall quality improvement (QI) score for each study. All criteria were equally weighted, as the review aimed to balance architectural transparency, consent life cycle enforceability, regulatory alignment, and empirical maturity. While no studies were excluded solely based on quality, the assessment was used to weight the interpretation of results, identify recurring methodological limitations, and contextualize the strength of the evidence base. Quality assessment was performed independently by the authors SP and MB on all included studies, with disagreements resolved through a collective discussion to ensure consistency.

Aggregate quality trends were later analyzed to distinguish between conceptual proposals and empirically validated implementations and to support sensitivity analysis in the synthesis of results.

As part of this sensitivity analysis, findings were reexamined by restricting the synthesis to studies with QI ≥0.75. This analysis showed that while high-level architectural patterns (eg, hybrid on-chain or off-chain designs and smart contract–based consent modeling) remained consistent, claims related to automated revocation propagation, usability validation, and enforcement assurance were supported by only a small subset of higher-quality studies. No study reported quantitative latency or completeness guarantees for revocation enforcement across distributed data custodians.

## Results

### Overview

This section presents the findings of the systematic review based on the analysis of 55 peer-reviewed studies that explore blockchain-based consent management frameworks for health care data sharing and secondary data use. The results are structured following the PRISMA process and analyzed according to the 4 conceptual dimensions defined earlier: consent life cycle management, auditability and traceability, usability and patient empowerment, and legal and ethical alignment.

### Conceptual Overview

**Figure 1.**
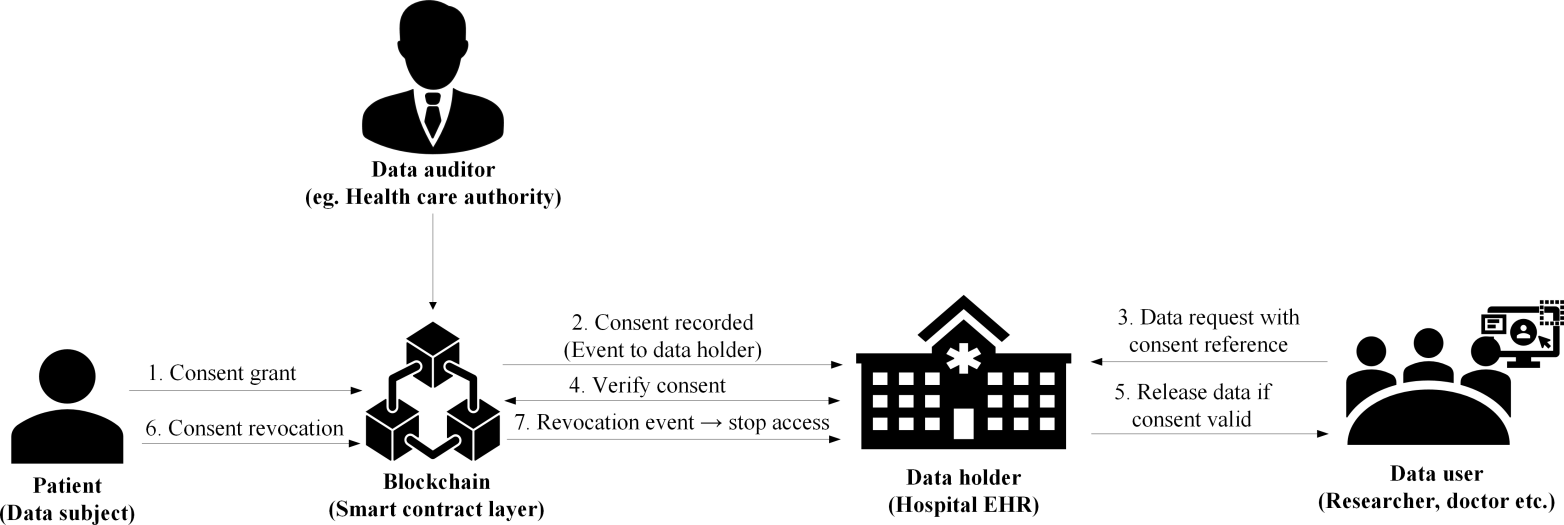
Conceptual architecture of blockchain-based consent management for secondary use of health data. EHR: electronic health record.

Before presenting the results of the systematic analysis, Figure 1 illustrates a generalized conceptual architecture reflecting the most common consent workflow patterns identified across the reviewed studies. The model summarizes how blockchain-based infrastructures support consent granting, verification, revocation, and audit logging for the secondary use of health data and serves as a reference architecture for the subsequent thematic analysis.

This conceptual model is not derived from a single implementation but represents an abstraction of recurring architectural elements observed across the literature, including hybrid on-chain or off-chain designs, smart contract–based consent logic, DIDs, and off-chain policy enforcement components. In line with the majority of reviewed frameworks, the model assumes a permissioned or consortium blockchain environment, in which participating health care and research institutions operate trusted nodes under defined governance and access control policies.

As shown in Figure 1, the consent workflow proceeds through the following steps.

- Step 1 begins when the data subject grants consent through a patient-facing application or digital identity wallet. This consent action is submitted to the blockchain via a smart contract, which records the consent state immutably on-chain.
- Step 2 propagates the consent creation or update event from the blockchain to the data holder, enabling synchronization of local access control and enforcement mechanisms.
- Step 3 occurs when a data requester submits a request for secondary use of health data, including a reference to the relevant consent identifier.
- Step 4 represents a bidirectional interaction between the data requester or data holder and the blockchain, in which the smart contract verifies the current consent state and returns an acknowledgment indicating whether access conditions are satisfied.
- Step 5 allows the data holder to release the requested data if consent has been verified and all access conditions are met. Sensitive health data remain off-chain throughout this process and are accessed only through controlled institutional interfaces.
- Step 6 enables the data subject to revoke previously granted consent, with the revocation recorded on-chain through a smart contract transaction.
- Step 7 disseminates the revocation event to subscribed data holders, ensuring that local enforcement mechanisms are promptly updated and that subsequent access requests are denied in accordance with the updated consent state.

Although the blockchain serves as the authoritative record of consent, data holders maintain a synchronized local enforcement state to support low-latency access control, auditing, and operational continuity. This local state is derived from on-chain consent events (steps 2 and 7) and does not replace blockchain-based verification.

Overall, blockchain functions primarily as a coordination and audit layer, anchoring consent events and access proofs rather than storing personal health data. This baseline hybrid architecture underpins the subsequent analysis of consent life cycle management, auditability, usability, and legal-ethical alignment across the reviewed studies.

### Study Selection

The comprehensive search across 3 academic databases (PubMed, Scopus, and Web of Science) initially yielded 205 records. After duplicate removal and screening for relevance, 55 (26.8%) studies met all inclusion criteria.

**Figure 2.**
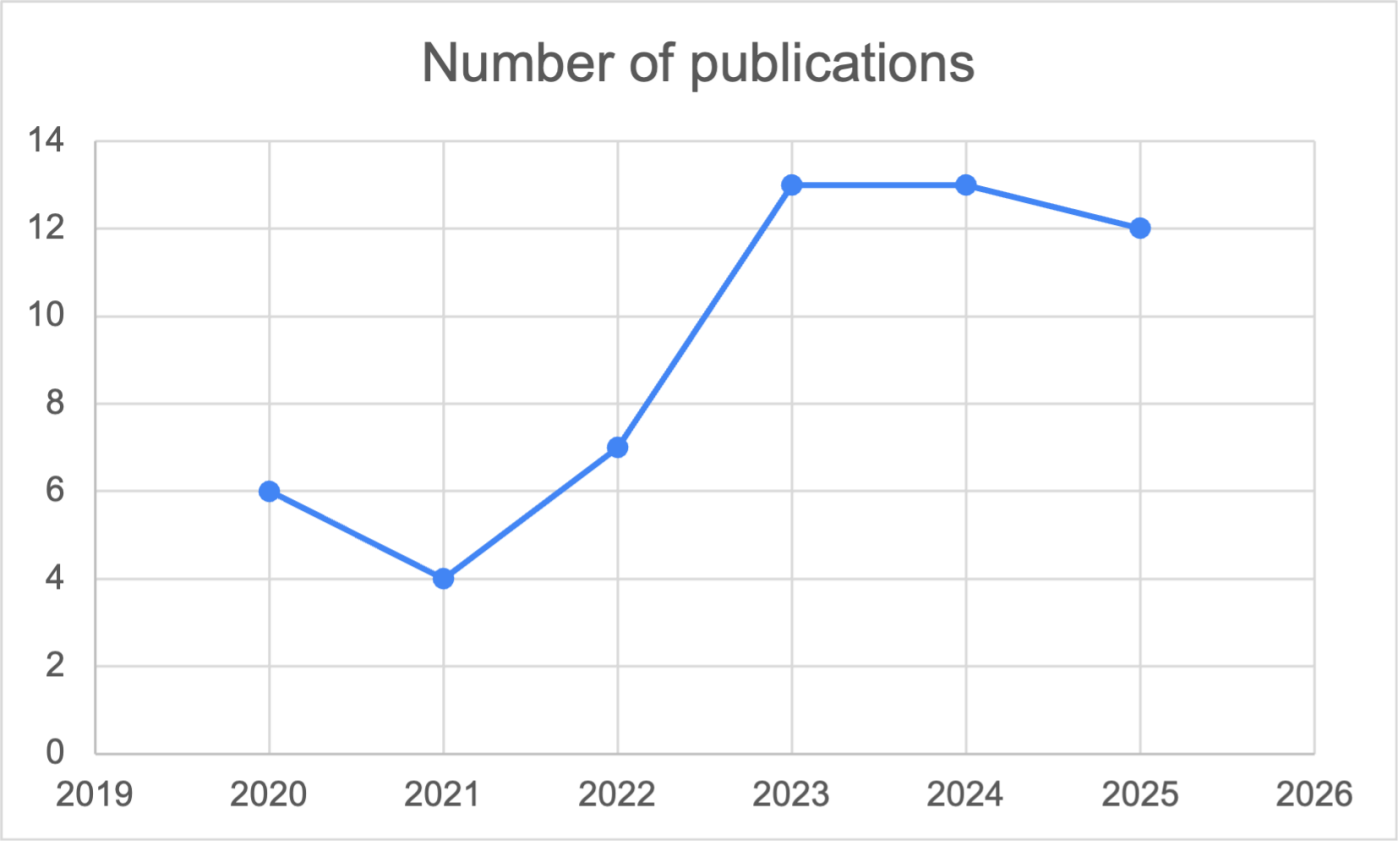
Publication trend of blockchain-based consent management studies (2020‐2025).

The included studies were published between 2020 and 2025, indicating that blockchain-enabled consent management is a relatively recent and rapidly expanding area of research. The number of publications peaked in 2023, reflecting the growing maturity and convergence of blockchain and health care data sharing technologies. Figure 2 shows the publication trend of the articles across the years.

**Figure 3.**
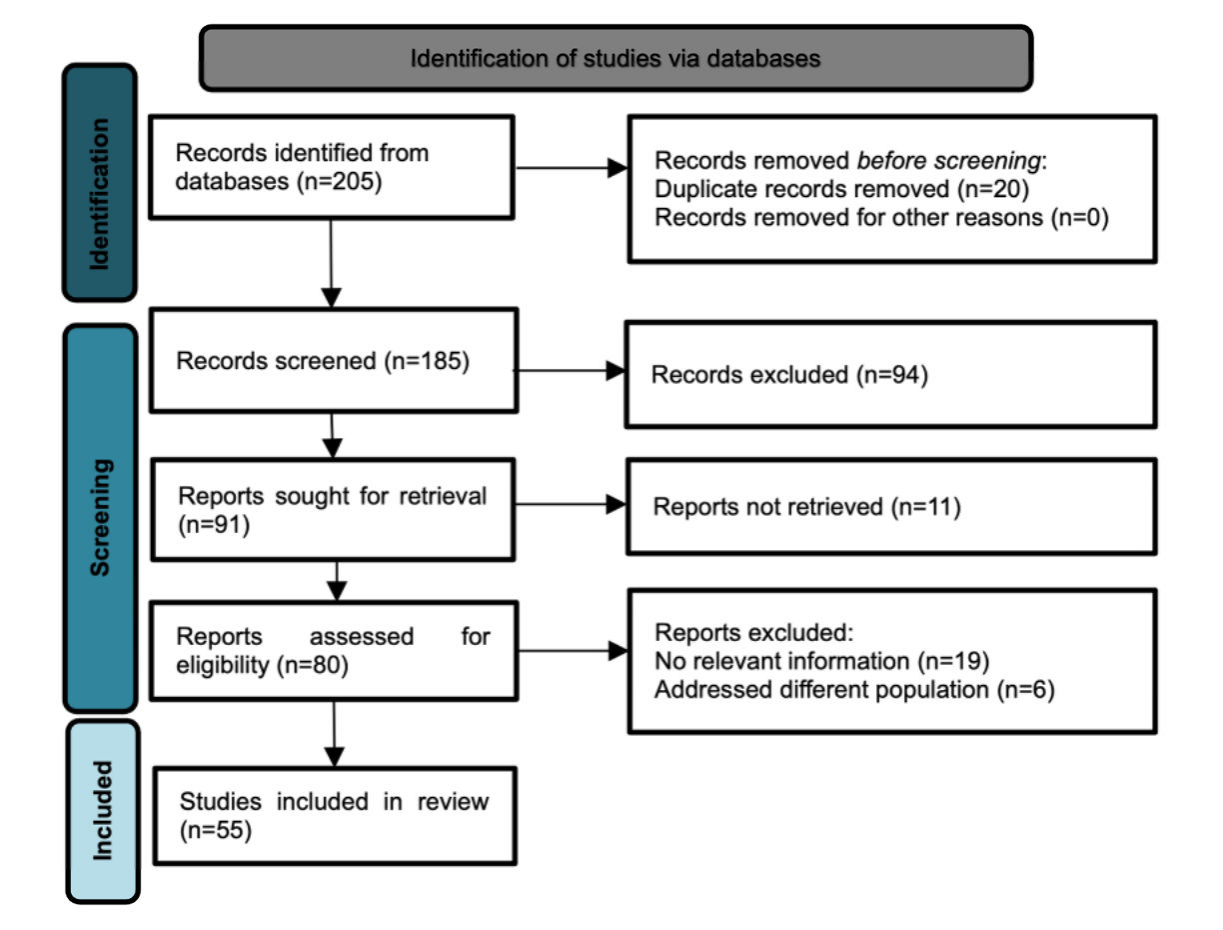
PRISMA (Preferred Reporting Items for Systematic Reviews and Meta-Analyses) workflow.

The PRISMA 2020 flow diagram presented in Figure 3 illustrates the identification, screening, eligibility assessment, and final inclusion of studies, while a detailed summary of all included studies is provided in Table 2.

Specifically, 205 records were identified through database searching. After removal of 20 (9.8%) duplicates, 185 (90.2%) records were screened by title and abstract, of which 94 (45.9%) were excluded. Ninety-one (44.4%) full-text articles were sought for retrieval, 11 (5.4%) could not be accessed, and 80 (39%) reports were assessed for eligibility. Of these, 25 (12.2%) were excluded for not meeting the inclusion criteria, resulting in 55 (26.8%) studies included in the final synthesis.

**Table 2.** Overview of quantitative characteristics of included studies.

Category	Dominant themes or counts
Total studies included	55
Year range	2020‐2025
Most active publication year	2023‐2024
Blockchain-based systems	43
Dynamic or revocable consent	39
Health care–related applications	52
Privacy or security addressed	55
Leading application areas	Clinical trials, PHRs^a^, digital health research

a PHR: personal health record.

### Descriptive Summary of Included Studies

Among the 55 papers, 43 (78%) explicitly implemented or proposed blockchain-based consent management frameworks, and 39 (71%) studies incorporated features of dynamic or revocable consent. Fifty-two (95%) studies addressed health care data sharing scenarios, and every study engaged with data security and privacy considerations.

The reviewed studies were thematically classified based on their primary analytical focus. Although several studies address multiple themes (as reflected in Table 2), each paper was assigned to one dominant category for the purpose of aggregation. The dominant category was determined based on the main contribution emphasized in the study’s objectives, system design, and evaluation. While this classification assigns a single dominant theme per study for summary purposes, Table 2 provides a multilabel thematic mapping, allowing studies to be associated with multiple categories. The category “Blockchain” refers to studies whose primary contribution lies in the design or analysis of blockchain-based systems or infrastructures, rather than in consent mechanisms or application-level workflows. Across the reviewed corpus, the largest proportion of studies addressed data security and privacy (n=55, 21.7%), followed by health care data sharing (n=52, 20.6%) and consent management (n=50, 19.8%). Blockchain-focused studies accounted for 17.0% (n=43), while dynamic consent represented 15.4% (n=39). Review articles constituted the smallest share at 5.5% (n=14).

In terms of domain focus, clinical trials accounted for the largest application area, followed by PHRs and digital health research environments. Fewer studies focused on biomedical research governance, genomic data sharing, or cross-institutional interoperability pilots.

Table 2 summarizes the quantitative characteristics of the included studies. The majority of works were published between 2022 and 2024, reflecting the rapid growth of interest in blockchain-enabled consent management. Ethereum and Hyperledger Fabric were the most frequently used blockchain platforms, while Quorum and Polygon appeared in several experimental prototypes, where the majority of reviewed systems were deployed on permissioned or consortium blockchains, reflecting governance and access control requirements in regulated health care environments.

### Influence of Application Domain, Platform Choice, and System Maturity

The reviewed studies exhibited substantial heterogeneity in application domain, blockchain platform, and implementation maturity, which influenced the nature and strength of reported findings. Studies focused on clinical trials and biomedical research more frequently implemented dynamic consent and auditability mechanisms, reflecting established governance requirements and sponsor accountability. In contrast, frameworks targeting EHRs and health information exchange more often emphasized access control and interoperability but provided less detail on consent life cycle enforcement.

Platform choice also affected system design. Permissioned and consortium blockchains, particularly Hyperledger Fabric, were predominantly used in health care–specific deployments and pilot studies, enabling finer governance control and alignment with regulatory requirements. Public blockchain–based proposals, often built on Ethereum, were more common in conceptual or proof-of-concept studies and tended to focus on smart contract expressiveness rather than operational compliance.

Finally, implementation maturity strongly shaped reported outcomes. Conceptual and prototype-level studies frequently claimed support for revocation and patient empowerment, whereas deployed or pilot systems more often reported constraints related to latency, institutional integration, and usability. As a result, higher-maturity implementations demonstrated narrower but more realistic functionality, while lower-maturity studies reported broader capabilities with limited empirical validation.

### Thematic Classification

The selected studies were systematically analyzed according to the 4 conceptual dimensions defined in the analytical framework: (1) consent life cycle management, (2) auditability and traceability, (3) usability and patient empowerment, and (4) legal and ethical alignment. This thematic synthesis reveals both the convergence of design strategies across studies and the heterogeneity in their technical maturity, regulatory focus, and evaluation depth.

### Consent Life Cycle Management

Across the dataset, most frameworks supported dynamic consent flows allowing users to grant, modify, and revoke consent at multiple stages of data sharing. Smart contracts were widely used to represent consent states, recording events such as *ConsentGranted*, *ConsentUpdated*, and *ConsentRevoked, which represent the consent states as the programmable states within the smart contracts, and they are automatically triggered based on predefined conditions*. Dynamic and revocable consent was the most frequently addressed theme across the dataset. Approximately two-thirds of the selected papers implemented explicit revocation mechanisms, with 35 (64%) of 55 studies distinguishing between active revocation (initiated by the data subject) and 6 (11%) of 55 papers adopting time-based or condition-based revocation (automatically triggered by contract logic). However, only 3 (5%) of 55 studies relied on administrator-initiated withdrawal.

To provide an overview of how consent life cycle management is addressed across the literature, Table 3 summarizes the studies that explicitly support dynamic consent features, including mechanisms for granting, modifying, and revoking consent. The table highlights the diversity of application domains and technical approaches through which life cycle control is operationalized in blockchain-enabled frameworks.

**Table 3.** Studies addressing consent life cycle management.

Authors and year	Major contribution	Application area
Kim et al (2022) [23]	Mobile PHR^a^ app with dynamic consent history (SUS^b^ validated)	PHRs
Alhajri et al (2022) [24]	Decentralized GDPR^c^-compliant consent via smart contracts	Wearable fitness data or health care
Albalwy et al (2021) [5]	Designed ConsentChain enabling patients to dynamically grant or withdraw consent for genomic data sharing; ontology-based automation of consent	Clinical genomic data sharing
Can et al (2024) [20]	Proposed hybrid architecture with a “purpose tree” for consent management	General GDPR consent management
Dong et al (2023) [25]	Built BEST platform with patient-controlled consent	PHRs
Jaiman and Urovi (2020) [26]	Created a dynamic consent model with DUO^d^+ADA-M^e^ ontologies, machine-readable consent, and revocation for health data	General health data sharing
Peyrone and Wichadakul (2023) [27]	Supported consent withdrawal, renewal, and audit logs	General data sharing
Daudén-Esmel et al (2024) [28]	Proposed a blockchain-based access control system for health care consent management	Health care consent and access control
Charles et al (2024) [19]	Presented blockchain-based dynamic consent model for patient-centric research, clinical trials, and health informatics	Patient-centric research and clinical trials
Roman-Martinez et al (2023) [29]	Proposed service-oriented blockchain architecture integrating consent management	Health care data sharing and consent management
Kim et al (2021) [30]	Built a patient-consent blockchain platform (PHR) using hyperledger fabric with on-/off-chain data storage	PHRs
Mishra and Mehra (2025) [31]	Proposed DiabeticChain, a blockchain framework for patient-controlled diabetes data sharing	Diabetes or chronic disease management
Pham et al (2024) [32]	Demonstrated feasibility of informed consent system for multisite clinical research dynamic consent handling across thousands of records	Clinical research
Albanese et al (2020) [15]	Proposed a private blockchain architecture to manage dynamic consent in clinical trials	Clinical trials
Muller et al (2023) [14]	Survey-based study showing patients prefer dynamic consent and continuous communication in large-scale data reuse	Large-scale health data reuse
Dankar et al (2020) [33]	Introduced dynamic informed consent for large-scale genomics; proposed dynamic consent as a solution for transparency and patient control	Genomics or population sequencing
Kim et al (2021) [34]	Designed Dynamichain, integrating dynamic consent for medical data sharing	Health care data ecosystem
Barnes et al (2025) [35]	Introduced “demonstrated consent”: a hybrid model combining blockchain (NFTs^f^ tied to samples) and generative AI^g^ (LLMs^h^) for interactive, transparent communication in biobanking	Biobanking
Khalid et al (2023) [36]	Proposed a formal security model for dynamic consent management system	Dynamic consent in e-health care
Huh et al (2022) [37]	Patient engagement with blockchain-enabled dynamic consent	Clinical trials
Despotou et al (2020) [38]	Surveyed patient attitudes on blockchain-based digital consent for dynamic health data sharing	Digital health or patient perception
Lee et al (2023) [39]	Empirical study identifying organizational, technical, and social factors that promote or hinder adoption of dynamic consent in digital health ecosystems	Digital health
Tith et al (2020) [40]	Developed purpose-based access control model for patient consent	EHRs^i^ or biobanking
Khalid et al (2023) [41]	Proposed decentralized privacy-first dynamic consent management system with patient empowerment	e-Health care
Singh and Rathee (2025) [42]	Developed a smart contract–based dynamic consent model integrating decentralized storage (IPFS^j^) and fine-grained access control for health care data	Health care applications
Rohini et al (2024) [43]	Introduced a blockchain-based consent model for Health Information Exchange	Health information exchange
Lee and Lee (2022) [18]	Dynamic consent in IoT^k^ health care	Health care IoT
Castro et al (2024) [44]	Identifies patient consent management as a core application of blockchain in clinical trials, highlighting smart contracts for automating electronic and dynamic consent	Blockchain-enabled consent management
Gondode et al (2025) [45]	Highlights blockchain-based management of patient consent and advance directives in critical care, using immutable records and smart contracts	Patient consent and ICU^l^ and emergency care
Gupta et al (2024) [46]	Highlights blockchain-based informed consent in clinical trials as a critical use case, emphasizing smart contracts for recording, reconsenting after protocol changes	Blockchain-enabled dynamic consent management
Kasyapa and Vanmathi (2024) [47]	Blockchain-based patient control and consent mechanisms using smart contracts, emphasizing secure authorization, patient ownership of EHRs, automated access control, and dynamic consent handling	Patient-centric consent and access control
Nguyen et al (2025) [48]	Proposes a dual-blockchain ecosystem that enables fine-grained, segment-level access control over DNA sequences, allowing patients to dynamically assign, update, and revoke permissions	Dynamic consent and access control
Righi et al (2025) [49]	Incorporates GDPR-compliant informed consent within edge controllers, allowing citizens to subscribe to health services, select which vital signs to share, define data usage purposes, and revoke participation by deleting accounts, supporting user-controlled and dynamic consent in large-scale monitoring systems	User-driven consent and access control
Felemban et al (2025) [50]	Highlights blockchain-enabled dynamic and surgical consent models, proposing decentralized, timestamped, and patient-accessible consent records	Dynamic informed consent for surgery
Hovorushchenko et al (2023) [51]	Physicians request access through validators and custodians, supporting consent-driven authorization, role-based access, and controlled release of medical data using smart contract–like workflows	Consent-aware sharing of electronic medical data

a PHR: personal health record.

b SUS: System Usability Scale.

c GDPR: General Data Protection Regulation.

d DUO: Data Use Ontology.

e ADA-M: Automatable Discovery and Access Matrix.

f NFT: nonfungible token.

g AI: artificial intelligence.

h LLM: large language model.

i EHR: electronic health record.

j IPFS: InterPlanetary File System.

k IoT: Internet of Things.

l ICU: intensive care unit.

To examine revocation propagation mechanisms in greater depth, Table 4 presents a focused analysis of representative frameworks, detailing their propagation mechanisms, enforcement layers, operational scope, and evaluation evidence.

For example, DynamiChain [34] enabled real-time consent expiry via time stamp validation, and the works presented by [5,32,40] also used Solidity contracts to encode purpose-specific permissions and time-bound access. While some other works, such as [25,34], represented consent through chaincode with endorsement policies ensuring institutional accountability. The works such as METORY [52] and BEST [25] demonstrated practical synchronization between blockchain and off-chain access control layers. While few works, such as [20,29], explored how consent withdrawal could automatically cascade to all data custodians through event listeners or application programming interfaces. Overall, most studies successfully demonstrated programmable consent logic at a conceptual or prototype level, but large-scale validation in production environments remained limited.

**Table 4.** Revocation propagation: mechanism, scope, and evidence.

Study	Propagation mechanism	Enforcement layer	Scope	Evaluation	Latency or assurance reported	Notes
DynamiChain (Kim et al, 2021) [34]	NR^a^ (conceptual time-bound expiry; no explicit listener or API^b^ described)	On-chain check (time stamp validation)	NR (likely single site or conceptual)	Sim or laboratory (conceptual prototype)	NR	Implements consent expiry via time stamps; propagation beyond a single relying party appears conceptual only
BEST (Dong et al, 2023) [25]	Event listener or API integration to off-chain access control (described)	Hybrid (blockchain audit+off-chain PDP^c^/PEP^d^)	Single site (NR for cross-institution)	Laboratory prototype	NR	Demonstrates synchronization between ledger events and PHR^e^ sharing controllers; no explicit latency benchmarks
METORY (Huh et al, 2022) [52]	Event listener or API (NR for exact pattern)	Hybrid (smart contract status+site controllers)	Multisite (clinical trial settings)	Pilot (multicenter)	NR	Operational deployment in multicenter trials; improved transparency or retention reported; no latency numbers
Roman-Martinez et al, 2023 [29]	Service orchestration or API gateway (conceptual)	Hybrid (on-chain state+off-chain services)	Cross-institution (conceptual)	Conceptual or laboratory	NR	Architecture-level description; propagation pathways proposed but not benchmarked (conceptual only)
Can et al, 2024 [20]	Event listeners or APIs (described; implementation depth NR)	Hybrid (auditable consent with on-/off-chain)	Multisite (NR for real deployment)	Laboratory or prototype	NNR	Hybrid auditable consent; propagation discussed; evidence suggests prototype-level implementation without latency metrics

a NR: not reported.

b API: application programming interface.

c PDP: policy decision point.

d PEP: policy enforcement point.

e PHR: personal health record.

Only 5 (9%) of the 55 reviewed studies described any mechanism intended to propagate consent revocation beyond the blockchain layer. Among these, only 3 (5%) studies implemented or evaluated such propagation beyond a purely conceptual design. None quantified propagation latency. After sensitivity analysis (QI ≥0.75), this reduced to 0 studies, underscoring the critical gap in real-world implementation of revocable consent (Table 4).

Beyond the low prevalence of revocation propagation mechanisms, the reviewed studies provide limited insight into the underlying reasons for this gap. Across the literature, revocation is typically modeled as a smart-contract state transition, while downstream enforcement across distributed off-chain systems is left implicit. This suggests unresolved technical challenges in synchronizing on-chain consent states with heterogeneous institutional PEPs. In addition, regulatory uncertainty regarding the interpretation of “logical deletion” under GDPR Article 17, combined with the absence of performance benchmarks and certification incentives, appears to discourage implementation beyond proof-of-concept designs.

### Auditability and Traceability

Auditability was one of the most consistent themes across the corpus. Blockchain’s immutability and append-only structure make it a natural foundation for verifiable audit trails. Among the reviewed studies, 27 (49%) of the 55 studies leveraged blockchain’s immutable ledger to maintain verifiable audit trails of consent and data access events, as summarized in Table 5.

**Table 5.** Studies focusing on auditability and traceability.

Authors and year	Audit or trace contribution	Application area
Can et al (2024) [20]	Hybrid architecture with consent management and introduced auditing mechanisms	Consent management
Dong et al (2023) [25]	Immutable logs in the proposed BEST platform	PHRs^a^
Peyrone and Wichadakul (2023) [27]	Supported consent withdrawal, renewal, and audit logs	General health care data sharing
Hang et al (2022) [6]	Comprehensive survey of blockchain in clinical trials, consent traceability, monitoring, data management	Clinical trials
Baysal et al (2023) [7]	Conducted multivocal literature review mapping blockchain applications in health care	General health domain
Garcia et al (2022) [53]	Proposed a blockchain-based privacy-preserving data governance framework ensuring accountability, transparency, and compliance across multiple stakeholders	Multistakeholder data governance
Daudén-Esmel et al (2024) [28]	Proposed a blockchain-based access control system for health care consent management; emphasized efficiency, scalability, and secure patient-controlled access to medical records	Health care consent and access control
Roman-Martinez et al (2023) [29]	Proposed blockchain architecture integrating consent management, access control, and auditing for compliance and interoperability	Health care data sharing or consent management
Ali et al (2023) [54]	Proposed a hybrid blockchain–deep learning architecture for secure and scalable health care data management	General health care systems
Kim et al (2021) [30]	Built a patient-consent blockchain platform (PHR) using hyperledger fabric with on/off-chain data storage; immutable PHR logs	PHRs
Pham et al (2024) [32]	Demonstrated feasibility of immutability in access logs	Clinical research
Albanese et al (2020) [15]	Auditability in clinical trials	Clinical trials
Kim et al (2021) [34]	Designed Dynamichain, a blockchain-based health care ecosystem integrating dynamic consent for medical data sharing; demonstrated system architecture and use cases	Health care data ecosystem
Hovorushchenko et al (2023) [51]	Surveyed blockchain-based approaches for medical data management; proposed classification of methods, highlighting challenges in scalability, privacy, and interoperability	General health care data management
Huh et al (2022) [52]	Designed METORY platform, a blockchain-based DCMS^b^ tailored for clinical trials, supporting dynamic consent updates, secure auditability, and decentralized governance	Clinical trials
Tith et al (2020) [40]	Developed purpose-based access control model for patient consent, implemented hyperledger fabric audit trials	EHRs^c^ or biobanking
Goint et al (2023) [55]	Proposed a framework for securing off-chain data storage in blockchain-based consent systems	Health care data sharing or off-chain storage
Rohini et al (2024) [43]	Introduced a blockchain-based immutable logging	Health information exchange
Castro et al (2024) [44]	Demonstrates blockchain’s role in enhancing transparency, data integrity, traceability, and reproducibility of clinical trial data	Transparent and auditable clinical trial
Gondode et al (2025) [45]	Shows how blockchain enables tamper-proof EHRs, immutable audit trails, and transparent data sharing in ICUs^d^	Auditable EHR management
Gupta et al (2024) [46]	Synthesizes cross-industry evidence showing blockchain’s role in enabling immutable audit trails and end-to-end traceability	Auditable supply chain management
Kasyapa and Vanmathi (2024) [47]	Systematically synthesizes how blockchain enables immutable audit trails, data integrity, and end-to-end traceability across health care use cases	Auditable EHR systems
Merlec and In (2024) [56]	Analyzes how blockchain-integrated decentralized storage ensures immutable records, verifiable data transactions, cryptographic integrity	Auditable decentralized storage
Nguyen et al (2025) [48]	Demonstrates how immutable ledgers and encrypted on-chain access tickets provide tamper-proof audit trails	Auditable genomic data sharing
Righi et al (2025) [49]	Proposes a hierarchical edge-fog-cloud architecture with data traceability services, sharding-based storage, and immutable logging of vital sign data paths	Auditable remote health monitoring
Felemban et al (2025) [50]	Synthesizes current and emerging uses of blockchain for immutable audit trails and traceability in health care	Auditable EHR systems
Hovorushchenko et al (2023) [51]	Proposes blockchain-based methods for medical data management that ensure immutable storage, provenance, and tamper-proof audit trail	Auditable EHR management

a PHR: personal health record.

b DCMS: Dynamic Consent Management System.

c EHR: electronic health record.

d ICU: intensive care unit.

Three dominant audit models emerged:

1. On-chain logging, where all consent transactions are recorded directly on the ledger for maximum transparency.
2. Hybrid anchoring, where detailed logs are stored off-chain but cryptographically hashed and referenced on-chain.
3. Smart-contract state verification, where consent status is queryable through verifiable contract variables.

While these models enhance transparency, several studies noted that blockchain audit logs remain technically complex and not easily interpretable by patients or regulators. Only a small subset provided visual audit dashboards or user-readable verification tools, such as the works presented in METORY [52], BEST [25], and SmartDataTrust [27], which provided graphical summaries of consent events. However, most systems exposed raw transaction logs or hashes, which are not easily interpretable by patients or auditors. This indicates that technical auditability does not always translate to practical accountability.

A small subset, 7 (13%) of 55 studies such as [29,43], implemented FHIR AuditEvent resources to bridge blockchain logs with clinical systems. Some recent works, such as [41], are experimenting with zero-knowledge proofs to verify data access without revealing identifiers, which preserves the privacy of data accessors while exposing only the minimum information required for audit and compliance.

### Usability and Patient Empowerment

Usability remains a significant bottleneck in real-world adoption, and findings in this literature study were also mixed. Many systems incorporated patient-facing interfaces or portals that allowed individuals to view and adjust consent preferences. Altogether, 19 (35%) of 55 studies proposed some form of user interface for managing consent, yet only 11 (20%) of 55 studies conducted formal usability or acceptance testing, as summarized in Table 6. Most systems targeted patients via mobile or web dashboards, but interactions often required manual cryptographic signing or wallet operations beyond typical health literacy levels. Approximately one-third of the studies integrated decentralized identity wallets supporting DIDs and VCs to give users more direct control. Technical interactions, such as cryptographic signing and blockchain transactions, were often found to be too complex for typical patients. Where formal usability studies were conducted, results showed improved trust and willingness to share data when transparency and visual feedback mechanisms were present. Overall, patient empowerment was widely emphasized but poorly operationalized, revealing a significant gap between conceptual ideals and deployable user experiences.

**Table 6.** Studies emphasizing usability and patient empowerment.

Authors and date	Usability or empowerment focus	Application area
Kim et al (2022) [23]	Developed and tested a blockchain-applied mobile PHR^a^ app with usability validation (System Usability Scale score 74.0)	PHR
Charles et al (2024) [19]	Presented blockchain-based dynamic consent model for patient-centric research, clinical trials, and health informatics applications	Patient-centric research or clinical trials
Dewan et al (2025) [57]	Co-designed a decentralized mobile app (“de-bi”) with patients with breast cancer, enabling transparency and engagement in biobank specimen use	Biobanking or patient engagement
Schmidt et al (2025) [8]	Scoping review mapping technical implementations of informed consent in digital health; identified challenges (obtain, prove, retrace, and manage)	Digital health or mobile health consent
Kim et al (2021) [30]	Hyperledger Fabric with on/off-chain data storage; empowered patients with ownership of PHRs, dynamic consent, and secure sharing across hospitals	PHRs
Mishra and Mehra (2025) [31]	Proposed DiabeticChain, a blockchain framework for patient-controlled diabetes data sharing	Diabetes or chronic disease management
Muller et al (2023) [14]	Survey-based study showing patients prefer dynamic consent and continuous communication in large-scale data reuse; emphasized patient-centric governance	Large-Scale Health Data Reuse
Dankar et al (2020) [33]	Highlighted ethical challenges in sequencing and proposed dynamic consent as a solution for transparency and patient control	Genomics or population sequencing
Barnes et al (2025) [35]	Introduced “demonstrated consent”: a hybrid model combining blockchain (NFTs^b^ tied to samples) and generative AI^c^ (LLMs^d^) for interactive, transparent communication in biobanking	Biobanking
Despotou et al (2020) [38]	Surveyed patient attitudes on blockchain-based digital consent for dynamic health data sharing; found patients valued transparency, control, and security	Digital health or patient perception
Lee et al (2023) [39]	Empirical study identifying organizational, technical, and social factors that promote or hinder adoption of dynamic consent in digital health ecosystems	Digital health
Baines et al (2024) [58]	Systematic review on public willingness for secondary data use; found conditional support based on privacy, trust, dynamic consent, and clear benefit communication	Secondary use or public attitudes
Kassam et al (2023) [9]	State-of-the-art literature review on digital health consent from patient perspectives; found preferences for transparency, dynamic consent, and control over protected health information	Digital health
Cumyn et al (2023) [10]	Surveyed public preferences for transparency in secondary data use; highlighted demand for clear communication, control, and ethical oversight	Secondary use of health data
Rohini et al (2024) [43]	Introduced a blockchain-based consent model for HIE^e^, ensuring immutable logging, patient control, and streamlined data access	Health information exchange
Lee and Lee (2022) [18]	Conceptual framework for applying dynamic consent in pervasive health care, focusing on privacy-aware IoT^f^ and ubiquitous computing environments	Pervasive health care or IoT
Kazemzadeh (2025) [59]	Highlights patient-centered integration of AI by emphasizing informed consent and patient autonomy	Clinical workflows in ophthalmology
Malakar et al (2024) [60]	Explores patient empowerment through data ownership and control by assessing professionals’ views on patients’ rights to own, control, and decide use of their genomic data, emphasizing dynamic consent	Patient-centric genomic data management
Merlec and In (2024) [56]	Blockchain and smart contracts enable data self-sovereignty by giving users full ownership, control over access, sharing, and portability of their data, supporting user-centric and censorship-resistant data management	Self-sovereign decentralized storage

a PHR: personal health record.

b NFT: nonfungible token.

c AI: artificial intelligence.

d LLM: large language model.

e HIE: health information exchange.

f IoT: internet of things.

Of the studied articles, the mobile PHR app presented by Kim et al [23] achieved a System Usability Scale score of 74, while Dewan et al [57] co-designed the “de-bi,” biobanking app with patients, which resulted in enhanced trust and engagement, and the METORY platform by Huh et al [52] was found to have increased transparency and participant retention in multicenter trials.

Furthermore, 18 (33%) of 55 studies integrated DID or VC mechanisms for self-sovereign consent representation [19,41]. These frameworks aligned with emerging EUDI (European Digital Identity) Wallet specifications to enable portable, user-controlled consent tokens. Only a few works addressed multilanguage interfaces, visual consent explainers, or voice assistant features vital for inclusive health data governance. Studies that did incorporate co-design or participatory approaches [38,57] reported higher user trust and perceived autonomy. Collectively, while patient empowerment is a recurrent objective, the translation of this principle into intuitive design remains emerging. Human-centered and iterative design methods remain underused in this domain.

### Legal and Ethical Alignment

Compliance with privacy regulations such as GDPR and the EHDS was discussed in 19 of 55 (35%) studies. Most frameworks aimed to align with GDPR principles of informed and revocable consent (Article 7) and data minimization (Article 5). A recurring challenge was the conflict between blockchain immutability and GDPR’s right to erasure (Article 17). To address this, several systems used privacy-preserving designs, recording only hashed references or using off-chain pointers to revocable consent states. Only 9 of 55 (16%) studies addressed the “right to erasure” (Article 17) directly, highlighting the continuing tension between blockchain immutability and deletion requirements. Specifically, 12 of 55 (22%) studies used off-chain storage of personal data and on-chain hash references; 6 of 55 (11%) studies adopted revocable credentials or tokenized pseudonyms to enable logical deletion; and 3 of 55 (5%) studies explored zero-knowledge proofs to verify consent without revealing personal data [41], although these remained largely experimental.

The works presented in [19,49] linked blockchain consent to EHDS and EUDI wallet frameworks, indicating an emerging policy-driven research direction. Nonetheless, ethical analysis remained superficial in most papers, only 8 of 55 (15%) studies explicitly referenced bioethical principles, such as autonomy, justice, or beneficence.

Beyond such high-level alignment claims, concrete operational guidance for EHDS is now emerging. The TEHDAS guidelines for Health Data Access Bodies provide detailed recommendations on implementing opt-out from secondary use, including the establishment of national opt-out registries, citizen-facing portals, and cross-border recognition of opt-out choices. This underscores that EHDS compliance for blockchain-based consent systems requires integration with Health Data Access Bodies governance and institutional workflows, not only on-chain auditability or consent logic [3].

Furthermore, studies by van Drumpt [61] and Forster et al [62] provide a comprehensive legal analysis revealing that existing European Union (EU) data protection frameworks, including the GDPR and the EHDS Regulation, inadequately support the lawful and harmonized secondary use of health and genetic data across borders, and it advocates for new EU-level legislative measures to establish consistent legal bases for such data reuse. The work by Barnes et al [35] introduces the novel concept of “demonstrated consent,” a blockchain- and generative AI–based framework that uses nonfungible tokens (NFTs) and large language models to create a transparent, participant-centered system for managing, tracking, and communicating biobanking consent. In addition, a study by Zafar [63] provides a comprehensive analysis of the conflicts between blockchain’s immutability and data protection requirements, proposing a hybrid legal-technical framework that leverages off-chain storage, encryption, and privacy-enhancing technologies to achieve regulatory compliance while preserving blockchain’s core functionalities.

Thus, while legal alignment is widely acknowledged, comprehensive ethical governance and juridical interpretation of blockchain consent remain underdeveloped. Table 7 summarizes the studies that explicitly address legal and ethical alignment across these dimensions.

**Table 7.** Studies addressing legal and ethical alignment.

Authors and year	Legal or ethical focus	Application area
Alhajri et al (2022) [24]	Proposed a GDPR^a^-compliant, decentralized consent system for wearable fitness data using blockchain and smart contracts	Wearable fitness data or health care
Can et al (2024) [20]	Proposed a GDPR-compliant hybrid architecture with a “purpose tree” for consent management	General GDPR consent management (applied to health care)
Qu et al (2025) [64]	Proposed a blockchain-based EMR^b^ sharing method with ring signatures and off-chain storage; ensured security, privacy, transparency, and compliance with China’s PIPL^c^	EMR
Garcia et al (2022) [53]	Proposed a blockchain-based privacy-preserving data governance framework ensuring accountability, transparency, and compliance across multiple stakeholders	Multistakeholder data governance (health +cross-domain)
Schmidt et al (2025) [8]	Scoping review mapping technical implementations of informed consent in digital health; identified challenges (obtain, prove, retrace, manage), highlighted blockchain and dynamic consent as promising but underevaluated	Digital health or mHealth^d^ consent
Minssen et al (2020) [65]	Analyzed regulatory and legal challenges of data sharing in clinical trials under GDPR; focused on compliance, transparency, and secondary use of data	Clinical trials or legal and GDPR compliance
Pina et al (2024) [66]	Provided best practices for data minimization, anonymization, pseudonymization, encryption, and retention; applied case study in health care dataset; highlighted ethical and regulatory compliance	Database management and health care dataset
Vlahou et al (2021) [67]	Review of GDPR impacts on biomedical research and consent; called for harmonization of law and ethics, clarification of broad consent, and roadmap for secondary data use	Biomedical research governance
Muller et al (2023) [14]	Survey-based study showing patients prefer dynamic consent and continuous communication in large-scale data reuse; emphasized patient-centric governance	Large-scale health data reuse
Dankar et al (2020) [33]	Introduced dynamic informed consent for large-scale genomics; highlighted ethical challenges in sequencing and proposed dynamic consent as a solution for transparency and patient control	Genomics or population sequencing
Barnes et al (2025) [35]	Introduced “demonstrated consent”: a hybrid model combining blockchain (NFTs^e^ tied to samples) and generative AI^f^ (LLMs^g^) for interactive, transparent communication in biobanking	Biobanking
Khalid et al (2023) [36]	Proposed a formal security model for DCMS^h^; introduced definitions of confidentiality, availability, unforgeability, auditability; suggested integrating blockchain, differential privacy, and ZKPs^i^ to strengthen privacy-by-design	Dynamic consent in eHealth care
Becker et al (2024) [68]	Legal analysis of EU^j^ data protection law and ethical issues for secondary use of health or genetic data; argued current GDPR framework insufficient, recommending new legislation	Secondary use of health and genetic data
Baines et al (2024) [58]	Systematic review on public willingness for secondary data use; found conditional support based on privacy, trust, dynamic consent, and clear benefit communication	Secondary use or public attitudes
Cumyn et al (2023) [10]	Surveyed public preferences for transparency in secondary data use; highlighted demand for clear communication, control, and ethical oversight	Secondary use of health data
Khalid et al (2023) [41]	Proposed decentralized privacy-first DCMS with patient empowerment; used blockchain to decentralize consent and ensure transparency	eHealth care
Zafar (2025) [63]	Legal or technical review of conflicts between blockchain immutability and GDPR rights; suggested hybrid architectures and privacy-preserving techniques	Data protection or legal-tech
Kazemzadeh (2025) [59]	Provides a comprehensive ethical and regulatory analysis of AI in ophthalmology	AI in ophthalmology
Malakar et al (2024) [60]	Professional perspectives on patient data ownership, collective and laboratory ownership, copyright implications, GDPR-like privacy concerns	Data governance

a GDPR: General Data Protection Regulation.

b EMR: electronic medical record.

c PIPL: Personal Information Protection Law.

d mHealth: mobile health.

e NFT: nonfungible token.

f AI: artificial intelligence.

g LLM: large language model.

h DCMS: Dynamic Consent Management System.

i ZKP: zero-knowledge proofs.

j EU: European Union.

### Summary of Thematic Prevalence

To consolidate the thematic findings across the 55 reviewed studies, Table 8 summarizes the dominant design approaches, associated methodological characteristics, and proportional representation across the 4 analytical dimensions examined earlier. The purpose of this synthesis is to provide a holistic overview of how blockchain-based dynamic consent systems distribute their priorities between technical, usability, and regulatory domains and to indicate areas where further development and evaluation are needed.

**Table 8.** Summary of thematic prevalence across the reviewed studies.

Analytical dimension	Predominant approach	Studies, n (%)
Consent life cycle management	Smart contract–based grant/modify/revoke models	35 (64)
Auditability and traceability	Hybrid on/off-chain hash anchoring systems	27 (49)
Usability and empowerment	Web or mobile interfaces (with limited testing)	19 (35)
Legal and ethical alignment	GDPR^a^-compliant off-chain designs	19 (35)

a GDPR: General Data Protection Regulation.

These findings reflect a rapidly converging landscape in which blockchain technologies are increasingly adopted as trust anchors for consent tracking, audit logging, and integrity assurance. However, striking divergences remain in how thoroughly these patterns are implemented and evaluated. Most studies incorporate blockchain for immutable auditability, but fewer extend the evaluation to human-centered usability or cross-system enforcement of consent withdrawal. As detailed in the consent life cycle management analysis, revocation propagation beyond on-chain state updates remains rarely implemented and largely unvalidated in current blockchain-based consent frameworks.

To further clarify how recurring technical mechanisms align with European regulatory frameworks, Tables 9-11 disaggregate the previously consolidated analysis into goal-specific comparisons, focusing on data storage, consent and access enforcement, and auditability and interoperability, respectively. This restructuring enables direct comparison of how the same functional objectives are implemented across different blockchain-based consent frameworks and addresses the overlapping nature of design choices observed in the literature.

**Table 9.** Data storage and persistence strategies in blockchain-based consent frameworks.

Strategy	On-chain data	Typical use	Regulatory alignment	Key risk
Off-chain+hash anchoring	No	Integrity, audit	High	Hash linkage
Encrypted on-chain storage	Yes	PoC^a^ designs	Low	Erasure impossible

a PoC: proof of concept.

**Table 10.** Consent and access enforcement mechanisms for secondary use of health data.

Mechanism	Revocable	Enforcement scope	Limitation
Smart contracts+PEP^a^	Yes	Partial	Latency
Token-based consent	Yes	Weak	Cache staleness

a PEP: policy enforcement point.

**Table 11.** Auditability and interoperability approaches in blockchain-enabled consent systems.

Approach	Human readable	Standards aligned	Limitation
On-chain logs	No	Low	Poor usability
FHIR^a^ AuditEvent anchoring	Yes	High	Early adoption

a FHIR: Fast Healthcare Interoperability Resources.

Table 9 compares data storage and persistence strategies adopted by the reviewed studies. A clear convergence emerges toward off-chain storage combined with on-chain hash anchoring, which enables integrity verification and auditability while supporting data minimization and erasure requirements under GDPR Articles 5 and 17. Nevertheless, a small subset of studies, primarily proof-of-concept designs, propose storing encrypted health data or metadata directly on-chain. Such approaches raise significant regulatory and security concerns, particularly with respect to the right to erasure and long-term exposure risks, and remain misaligned with GDPR and EHDS expectations.

Table 10 focuses on consent and access enforcement mechanisms. Most reviewed frameworks rely on smart contracts combined with off-chain PEPs or token-based authorization to enable revocable and fine-grained access control. While these mechanisms support logical revocation under GDPR Article 7, enforcement is often partial and dependent on timely synchronization between the on-chain consent state and off-chain systems. As a result, revocation propagation delays and stale authorization caches remain recurring limitations across implementations.

Table 11 summarizes approaches to auditability and interoperability. Immutable on-chain logs are widely used to ensure nonrepudiation and traceability but are rarely human-readable or directly usable by regulators or patients. Emerging approaches that bridge blockchain events with health care standards such as HL7 FHIR Consent and AuditEvent resources demonstrate greater potential for institutional governance and EHDS-aligned interoperability, although adoption remains limited and implementations are still evolving.

Taken together, this goal-based analysis reinforces that blockchain is not inherently compliant or sufficient on its own. Regulatory alignment depends on how architectural components operationalize principles such as data minimization, revocability, accountability, and interoperability. Although architectural convergence is evident across storage and audit patterns, substantial gaps remain in enforcement assurance, usability validation, and governance integration.

### Quantitative Summary, Quality Assessment, and Emerging Trends

This section synthesizes the core quantitative findings from the 55 studies, evaluates their methodological quality, and highlights emerging architectures and research trajectories in blockchain-based dynamic consent for secondary health data use, with particular attention to how study quality influences the strength of reported design claims.

As discussed in the Consent Life Cycle Management analysis, sensitivity analysis restricted to higher-quality studies (QI ≥0.75) further confirms that operational revocation propagation beyond on-chain state changes remains rarely implemented and empirically unvalidated. Similarly, the proportion of studies reporting formal usability evaluation remained below 25%. Core architectural trends, such as hybrid on-chain or off-chain storage and smart contract–based consent modeling, remained consistent across quality thresholds. This indicates that while foundational design patterns are robust, claims related to operational enforceability and real-world readiness are supported by a limited subset of higher-quality studies.

### Quantitative Overview

A structured synthesis of the extracted data reveals strong convergence on several foundational design patterns for blockchain-based consent management. Table 12 presents the operational definitions and associated aggregate counts derived during quantitative coding.

**Table 12.** Operational definitions and counts used in the quantitative synthesis.

Metric	Operational definition	Count (n/N)	Percent
Blockchain consent system	Paper proposes or evaluates a consent framework implemented on blockchain or DLT^a^ with executable consent logic or on-chain logging.	43/55	78.2
Dynamic or revocable consent	Framework allows post hoc modification and/or withdrawal of consent enforced by smart contracts or off-chain controllers (not merely discussed conceptually).	39/55	70.9
User interface present	Prototype or product UI^b^ shown for patients/stakeholders to manage consent.	16/55	29.1
Formal usability testing	Reports a recognized method (eg, SUS^c^/UEQ^d^/think-aloud) with N and procedure.	11/55	20
Auditability implemented	On-chain or hybrid logging built, not only proposed.	18/55	32.7

a DLT: distributed ledger technology.

b UI: user interface.

c SUS: System Usability Scale.

d UEQ: user experience questionnaire.

These proportions reflect a strong trend toward technical feasibility and decentralized audit trails, with less attention to user-facing evaluative studies. In particular, fewer than 1 in 5 studies (<20%) report user experience validation, despite claims of user empowerment and transparency.

Publication trends from 2020 to 2025 show an accelerating interest in the topic, with a marked surge beginning in 2022. This aligns with regulatory movement around the EHDS and the maturation of decentralized identity standards, such as W3C DIDs and VCs.

### Quality and Agreement Assessment

To evaluate the reliability and robustness of the reviewed studies, a 5-item QI was applied across all papers, scoring on clarity of objectives, architectural transparency, consent life cycle coverage, privacy or regulatory alignment, and empirical validation. The resulting QI scores ranged from 0.35 to 0.95, with a median of 0.68 (IQR 0.54‐0.82), indicating overall moderate to high methodological quality.

- Interrater agreement was substantial (κ=0.78, range 0.71‐0.85).
- Excluding studies with QI <0.50 slightly reduced thematic counts (eg, dynamic consent: from 39 to 33) but did not reverse key trends.
- Notably, automated revocation propagation across system boundaries remains rare and empirically unvalidated, with no studies reporting latency or enforcement assurance metrics, representing an operational gap with direct implications for GDPR compliance (Article 7).

Beyond reporting agreement, the quality assessment provides critical interpretive context for the thematic synthesis. Studies with higher QI scores consistently offered clearer architectural descriptions, explicit consent-state models, and at least partial empirical validation, whereas lower-QI studies were more likely to present conceptual frameworks without implementation detail or reproducible evaluation.

When restricting the analysis to studies with QI ≥0.75, several commonly reported capabilities, particularly around real-time revocation propagation and user empowerment, were supported by only a very small subset of works, indicating that these features remain largely aspirational rather than operationally demonstrated in the current literature.

Similarly, claims of GDPR or EHDS compliance in lower-QI studies were often based on high-level alignment statements rather than concrete legal-technical mechanisms (eg, explicit handling of Article 17 erasure or cross-system enforcement), whereas higher-QI studies more frequently implemented hybrid storage, revocable credentials, or auditable consent-state transitions.

Taken together, these results emphasize that while the foundational architectural patterns for blockchain-enabled dynamic consent are maturing, the empirical depth needed to substantiate performance, usability, and regulatory enforcement remains limited. Evaluation rigor, latency benchmarking, and cross-institutional enforcement assurance therefore represent critical priorities for advancing from conceptual feasibility toward operational readiness.

Overall, the QI analysis suggests that the field is characterized by strong architectural innovation but uneven methodological maturity and that future research must prioritize reproducible implementations, measurable performance indicators, and user-centered validation to strengthen the evidence base for blockchain-based consent systems in real health care settings.

### Emerging Research and Technical Trends

Several emergent clusters signal the evolving priorities and directions in the field.

#### Self-Sovereign Consent With DIDs and VCs

There is increasing adoption of decentralized identity credentials to enable user-controlled consent traces and cross-domain portability, especially in cross-border care scenarios [49].

This trend reflects a shift from institution-centric consent registries toward patient-held, cryptographically verifiable consent artifacts, aligning with emerging EUDI Wallet and W3C VC ecosystems. However, only a limited subset of studies demonstrated live wallet integration or interoperability across domains, indicating that most implementations remain at prototype or conceptual stages.

#### FHIR and HL7-Aligned Interoperability

There is an increasing emphasis on bridging blockchain with clinical standards (eg, FHIR Consent and AuditEvent), in preparation for EHDS and HealthData@EU infrastructures [69].

Recent works explicitly map smart-contract consent states to FHIR Consent resources and expose blockchain audit events as FHIR AuditEvent records, signaling a growing recognition that blockchain layers must integrate with existing health IT workflows rather than operate as parallel infrastructures. Nonetheless, most proposals stop short of demonstrating production-grade interoperability or conformance testing against national FHIR profiles.

#### Privacy-Preserving Cryptography

There is experimental use of zero-knowledge proofs and selective disclosure schemes for authorization without identifier exposure, aligned with GDPR Article 25.

These approaches aim to reconcile blockchain transparency with data minimization by enabling verification of consent or access rights without revealing identities or attributes. However, implementations remain limited to proof-of-concept circuits, with unresolved challenges around computational overhead, verifier scalability, and standardization across platforms.

#### Hybrid Blockchain Architectures

The use of off-chain storage with on-chain hashes or audit indices to balance GDPR erasure rights with audit integrity has been a dominant architectural pattern since early blockchain-based health data exchange systems. Rather than an emerging trend, recent studies refine this baseline by introducing richer metadata anchoring, revocable pointers, and tighter coupling with off-chain PEPs, reflecting a shift from architectural choice toward operational optimization.

#### Policy-Driven Design

Many systems explicitly frame their architecture in terms of GDPR and EHDS compliance, emphasizing revocability, auditability, and patient agency.

This has led to architectures that embed legal concepts such as purpose limitation, consent expiry, and accountability directly into smart-contract logic. Nevertheless, legal alignment is often asserted rather than empirically validated, and few studies evaluate whether implemented mechanisms would satisfy regulatory scrutiny in real enforcement scenarios.

Across these trends, a common pattern emerges: architectural sophistication is increasing, but empirical validation and cross-system interoperability lag behind. Higher-quality studies (QI ≥0.75) are more likely to integrate multiple trends simultaneously, such as DIDs with FHIR bridging and hybrid storage, suggesting that technical convergence is occurring primarily in the most methodologically mature works. Together, these trends indicate a gradual transition from conceptual feasibility toward policy-aware and interoperability-oriented design. However, significant gaps persist in enforcement interoperability, large-scale deployment evidence, usability testing, and latency assurance, underscoring that most frameworks remain preoperational despite increasing architectural convergence.

## Discussion

### Summary of Main Findings

This systematic review analyzed 55 peer-reviewed studies published between 2020 and 2025 that proposed or evaluated blockchain-based frameworks for dynamic and revocable consent in the secondary use of health data. The findings indicate that most proposed systems leverage smart contracts and hybrid on-chain or off-chain architectures to support consent life cycle management and immutable auditability. While dynamic consent and revocation are frequently claimed features, only a small subset of studies provided architectural detail or empirical evidence demonstrating automated revocation propagation across distributed systems. Usability evaluation and real-world deployment remain limited, with fewer than one-fifth of studies reporting formal user testing. Furthermore, although GDPR alignment is commonly asserted, explicit consideration of the EHDS governance model and operational opt-out mechanisms is still sparse.

Sensitivity analysis based on methodological quality (QI ≥0.75) confirmed that high-level architectural patterns remain stable, whereas evidence for automated revocation propagation, usability validation, and enforcement assurance is limited to a small subset of higher-quality studies.

### Dynamic and Revocable Consent Life Cycle Management

In relation to the first objective of this review, characterizing architectural and operational approaches to dynamic and revocable consent, blockchain was widely adopted as an enabling infrastructure for managing dynamic and revocable consent life cycles in health care. Most frameworks used smart contracts to automate consent processes, recording events such as consent granting, modification, and revocation, and defining granular access conditions including time-bound or purpose-specific authorizations. By encoding consent states on-chain, these systems leveraged blockchain’s immutability to maintain verifiable histories of consent actions, conceptually aligning with principles of patient autonomy and ongoing informational self-determination.

However, despite frequent claims of revocability, the practical realization of dynamic consent remained limited. Only a small fraction of studies operationalized real-time revocation propagation across distributed data custodians, and many implementations relied on simulated environments rather than live data sharing scenarios. Even among higher-quality studies, revocation was typically enforced through local policy decision points querying on-chain consent state, rather than through end-to-end automated propagation across institutional boundaries. As a result, most frameworks achieved logical revocability at the ledger level but did not demonstrate effective enforcement across heterogeneous, off-chain data environments.

The increasing integration of DIDs and VCs reflects a broader shift toward self-sovereign consent models, enabling individuals to assert and manage permissions independently of central authorities. Nevertheless, most DID- and VC-enabled systems stopped short of demonstrating cross-domain portability or wallet-to-wallet interoperability, indicating that self-sovereign consent remains largely aspirational. From a regulatory perspective, this gap is significant, as both the GDPR and the EHDS require not only the possibility of consent withdrawal but also its practical and timely effect on ongoing and future data processing.

The limited operationalization of revocation propagation observed in this review reflects a combination of technical, regulatory, and organizational factors rather than a lack of conceptual awareness. From a technical perspective, propagating consent withdrawal across distributed data custodians requires reliable event dissemination, low-latency synchronization, and tight coupling between blockchain layers and off-chain access control systems, capabilities that are difficult to implement and evaluate in academic prototypes. From a regulatory standpoint, uncertainty persists regarding how blockchain-based “logical deletion” mechanisms are interpreted under GDPR Article 17 and how these mechanisms will align with the EHDS governance model, particularly in federated infrastructures such as HealthData@EU. Organizationally, many proposed systems lack clear institutional incentives or compliance drivers to validate revocation enforcement beyond the ledger level, resulting in designs that prioritize auditability over enforceability. Together, these factors help explain why revocation is widely claimed but rarely demonstrated as an end-to-end operational capability.

### Auditability, Provenance, and Accountability

Addressing the second objective of assessing auditability and accountability mechanisms, auditability and provenance tracking emerged as among the most consistently addressed features of blockchain-based consent frameworks. Many studies capitalized on blockchain’s tamper-evident recordkeeping to provide verifiable logs of consent events and data access transactions, underpinning accountability in multi-institutional and cross-border data reuse scenarios. Three dominant design patterns were observed: direct on-chain audit logging, hybrid architectures anchoring off-chain logs on-chain, and smart contract state verification through read-only queries.

Hybrid audit models were the most prevalent, reflecting a balance between transparency, privacy, and scalability. These approaches typically stored detailed audit metadata off-chain while anchoring cryptographic proofs on-chain, allowing integrity and nonrepudiation without exposing sensitive identifiers. Such configurations are particularly relevant in GDPR and EHDS contexts, where data minimization and controlled disclosure are essential. Architecturally, this trend also coincides with a move away from public, permissionless blockchains toward permissioned or consortium-based ledgers governed by trusted health care or research institutions.

Despite strong technical guarantees, auditability often did not translate into practical accountability. Only a limited number of studies provided user-facing audit dashboards or regulator-oriented reporting tools, leaving audit data opaque to nontechnical stakeholders. In many cases, audit logs were verifiable by machines but inaccessible or unintelligible to patients, ethics committees, or oversight authorities. This highlights that accountability is not solely a cryptographic property but a sociotechnical one, requiring human-readable representations and institutional governance interfaces.

Emerging efforts to integrate blockchain logs with established health care standards, such as HL7 FHIR AuditEvent resources, represent a promising direction. However, these remain early-stage, and standardized frameworks for consent provenance visualization and regulatory reporting are still under development.

### Usability, Interoperability, and Operational Constraints

With respect to the objective of evaluating usability, interoperability, and implementation maturity, usability and real-world adoption remain persistent challenges. Only a minority of studies reported formal usability testing or user experience evaluation, and many systems required interactions with cryptographic wallets, private keys, or blockchain transactions that exceed typical patient or clinician digital literacy. These challenge assumptions underlying self-sovereign models and underscore the need for human-centered design approaches.

Frameworks incorporating mobile-friendly consent dashboards or DID wallets demonstrated greater potential for user acceptance, yet accessibility barriers such as limited multilingual support, unclear consent visualization, and insufficient mobile optimization were common. Studies with higher methodological quality were more likely to report participatory design or usability validation, reinforcing the link between technical maturity and user-centered evaluation.

Interoperability with existing health information systems also emerged as a critical bottleneck. Although several studies referenced integration with EHRs or HL7 FHIR resources, few described concrete mechanisms for synchronizing blockchain-based consent states with institutional access control systems or cross-border infrastructures. Without standardized interfaces and data models, blockchain-based consent solutions risk operating in isolation from clinical and research workflows, limiting their production viability. Increasingly, blockchain appears best positioned as a coordination and trust layer rather than a replacement for established health IT systems.

Scalability and performance concerns further constrain deployment. Most frameworks adopted permissioned ledgers or layer-2 solutions to address throughput and cost limitations, yet latency in consent updates and revocation propagation remains largely unmeasured. The near absence of reported performance benchmarks suggests that scalability is often assumed rather than empirically validated.

### Regulatory Alignment and Ethical Governance

Most reviewed studies explicitly referenced compliance with the GDPR, particularly requirements for informed and revocable consent. However, alignment was more often asserted than operationalized. The tension between blockchain immutability and rights such as erasure remains unresolved in many designs, with mitigation strategies such as off-chain storage, revocable credentials, or zero-knowledge proofs still largely experimental.

With the EHDS Regulation establishing dedicated governance structures for secondary use of health data in the EU, future consent architectures must increasingly move beyond GDPR-centric interpretations. Compliance under the EHDS depends not only on technical design but also on integration with HealthData@EU infrastructures, certification schemes, and institutional access bodies. Ethical governance was comparatively underdeveloped across the literature, with limited engagement with principles such as justice, beneficence, or the ethical implications of automated consent enforcement.

### Comparison With Existing Reviews

Several prior reviews have examined blockchain applications in health care or digital consent from complementary perspectives; however, their analytical scope differs substantially from that of this review. Surveys focusing on blockchain in health care have primarily emphasized data security, interoperability, or application domains such as clinical trials and supply chains, without systematically analyzing consent as a dynamic and revocable life cycle with enforceable propagation mechanisms. Conversely, reviews of digital or dynamic consent have largely centered on ethical, legal, or usability considerations, often without examining the underlying technical architectures required to ensure verifiable auditability and distributed enforcement.

In contrast to these earlier works, this review explicitly integrates architectural, operational, and regulatory dimensions by examining how blockchain-based frameworks implement consent life cycle management, auditability, usability, and legal alignment for secondary use of health data. By synthesizing evidence from 55 peer-reviewed studies published between 2020 and 2025, this review advances prior literature in 3 key ways. First, it systematically analyzes revocation enforcement and propagation mechanisms, revealing that revocability is frequently asserted but rarely operationalized across distributed data custodians. Second, it evaluates auditability not only as a cryptographic property but also as a sociotechnical capability requiring interpretability and governance integration. Third, it situates blockchain-based consent frameworks within the emerging EHDS regulatory context, which has been largely absent from previous reviews.

By consolidating fragmented evidence across technical, usability, and regulatory dimensions, this review moves beyond descriptive surveys and provides an integrative assessment of the operational maturity of blockchain-enabled consent systems. This positioning clarifies both the current limitations of existing approaches and the concrete design and governance challenges that must be addressed to enable real-world deployment for secondary use of health data.

Positioning this review within the broader consent literature reveals a clear progression in research focus. Reviews of dynamic consent outside blockchain contexts have predominantly examined ethical acceptability, user engagement, and governance models, often assuming centralized infrastructures and institutional enforcement. Conversely, surveys of blockchain in health care have focused on data security, provenance, and interoperability, treating consent as a secondary or implicit function rather than as a programmable life cycle.

This review differs by explicitly integrating these trajectories. By analyzing consent as an enforceable life cycle implemented through blockchain-based architectures, the review links ethical expectations of dynamic consent with the technical mechanisms required to operationalize revocation, auditability, and accountability in distributed secondary-use settings. In doing so, it exposes a persistent gap between conceptual consent models and their technical realization, particularly in relation to revocation propagation and cross-institutional enforcement, an aspect not systematically examined in earlier reviews.

### Implications for Design, Governance, and Policy

The findings suggest that future progress depends less on novel blockchain primitives and more on integrative system design. Blockchain should be treated primarily as a coordination and accountability layer rather than as a data repository. Effective systems must tightly couple on-chain consent states with off-chain PEPs, adopt interoperable standards such as HL7 FHIR for consent and audit representation, and embed legal interpretation into system governance.

From an institutional perspective, consortium-based deployments with clear roles and accountability mechanisms are better aligned with secondary-use contexts than public infrastructures. For policymakers and standards bodies, clearer guidance is needed on how blockchain-enabled consent can interoperate with EHDS governance models, including HealthData@EU and Health Data Access Bodies.

The observed variation across application domains, blockchain platforms, and system maturity suggests that blockchain-enabled consent solutions are not uniform and should not be evaluated as a single class of systems. Design trade-offs that are acceptable in clinical trial environments, such as controlled participation and sponsor oversight, may not translate directly to large-scale EHR or population health infrastructures. Similarly, while public blockchains support rapid prototyping and transparency, permissioned and consortium deployments appear more compatible with regulated secondary-use contexts under GDPR and the EHDS. These differences underscore the need for domain-sensitive evaluation rather than one-size-fits-all architectural assumptions.

### Toward an Operational Research Agenda for Blockchain-Based Dynamic Consent

To move beyond high-level design principles, the findings of this review motivate a concrete research agenda focused on operational validation rather than architectural novelty. First, future work should prioritize the design and evaluation of a multisite revocation propagation protocol, in which consent withdrawal events recorded on-chain are automatically disseminated to heterogeneous off-chain policy PEPs operated by independent institutions. Such a protocol should explicitly define event subscription mechanisms, enforcement acknowledgment semantics, and failure-handling procedures, enabling revocation to be evaluated as a measurable system property rather than a conceptual claim.

Second, pilot studies should report quantitative enforcement metrics, including revocation propagation latency, completeness of enforcement across data custodians, and system behavior under partial network or service failures. These metrics are directly relevant to GDPR Article 7 and EHDS requirements that withdrawal of consent have a timely and practical effect, yet they are absent from current implementations.

Third, human-centered design should be operationalized through participatory pilot deployments rather than interface descriptions alone. For example, wallet-based consent systems could be evaluated through longitudinal studies assessing user comprehension of consent states, revocation confidence, and perceived control when consent decisions have observable downstream effects.

Finally, interoperability claims should be validated through standards-based integration pilots, mapping smart-contract consent states to HL7 FHIR Consent and AuditEvent resources and testing end-to-end workflows within EHDS-aligned infrastructures such as HealthData@EU. Together, these directions define a transition from conceptual feasibility toward deployment-ready, regulatorily meaningful consent infrastructures.

### Future Research Directions

Future research should prioritize end-to-end revocation enforcement as a measurable system property, rather than treating revocation solely as an on-chain state transition. This includes the explicit design and evaluation of revocation propagation protocols that disseminate withdrawal events from blockchain ledgers to heterogeneous off-chain PEPs operated by independent data custodians. Such protocols should be evaluated using quantitative metrics, including propagation latency, enforcement completeness, and system behavior under partial failures.

In parallel, interoperability with EHDS-aligned infrastructures should move beyond architectural claims toward standards-based validation. Future studies should assess how blockchain-based consent states and audit events can be mapped to HL7 FHIR Consent and AuditEvent resources and exercised within cross-institutional workflows anticipated by HealthData@EU. These evaluations are essential to demonstrate regulatory readiness under the EHDS governance model.

Usability and patient empowerment should likewise be examined through longitudinal, user-centered evaluations rather than interface descriptions alone. In particular, future work should assess whether patients can meaningfully understand, manage, and trust consent withdrawal when revocation has observable downstream effects on data access. Wallet-based and credential-based consent models warrant participatory studies that measure comprehension, confidence, and perceived control over time.

Finally, advancing blockchain-enabled consent from prototype to practice will require interdisciplinary pilot deployments involving technologists, clinicians, legal scholars, ethicists, and policymakers. Such collaborations are necessary to align technical enforcement mechanisms with legal interpretation, institutional governance, and real-world operational constraints.

### Conclusions

This systematic review examined 55 peer-reviewed studies (2020‐2025) that propose or evaluate blockchain-enabled approaches to dynamic, auditable, and revocable consent for the secondary use of health data. The synthesis demonstrates clear architectural convergence: most systems rely on hybrid on-chain or off-chain designs, use smart contracts or equivalent logic to represent consent states, and use blockchain primarily as an integrity, coordination, and audit layer rather than as a storage substrate for sensitive health information. Many frameworks further integrate DIDs and VCs to bind consent states to portable identities and attributes, reinforcing the shift toward self-sovereign yet auditable consent artifacts.

Beyond documenting these trends, this review contributes novel insight by systematically evaluating consent as an enforceable life cycle rather than as a static design feature, linking architectural choices to revocation propagation, usability evidence, and regulatory readiness for secondary-use governance under the GDPR and the EHDS.

The evidence indicates that blockchain can substantively strengthen 2 aspects of secondary-use governance. First, it provides a shared, tamper-evident ledger for recording consent decisions and access events, improving verifiability and accountability across organizational boundaries. Second, it enables programmable consent modeling, granular permissions, time-bound authorizations, and policy-driven transitions, supporting the core intent of dynamic consent.

However, the review identifies a decisive gap between architectural feasibility and operational readiness. While revocation is widely claimed, most implementations demonstrate revocation only as an on-chain state transition or token invalidation, without validated propagation and enforcement across distributed off-chain systems. Sensitivity analysis restricted to higher-quality studies (QI ≥0.75) confirms that operational revocation enforcement is demonstrated by only a small subset of works, with none reporting quantitative guarantees for revocation latency or completeness across data custodians. From a regulatory standpoint, this is material: both GDPR and the EHDS framework require withdrawal to have practical and timely effect, not merely representational status, particularly in federated secondary-use infrastructures such as HealthData@EU.

A further cross-cutting finding is that technical auditability does not automatically translate into practical accountability. Many systems provide cryptographic proofs and transaction logs that are difficult for patients, auditors, and regulators to interpret. Moreover, despite frequent claims of patient empowerment, formal usability validation remains limited, and the cognitive burden associated with wallets, signing flows, and key custody continues to pose a barrier to real-world deployment.

Overall, this review demonstrates that while blockchain is a promising trust anchor for consent life cycle tracking and auditability in secondary use of health data, it is not inherently compliant or sufficient on its own. Regulatory alignment depends on the broader sociotechnical system, including enforcement mechanisms, interoperability with health IT infrastructures, governance arrangements, and user experience. Achieving operational maturity will therefore require governance-oriented design, quantitative evaluation of enforcement properties, and close integration with emerging European health data infrastructures.

This review advances the consent literature by reframing blockchain-based consent from a static compliance artifact to an enforceable, distributed life cycle subject to measurable system properties such as revocation latency, propagation completeness, and governance integration. Unlike prior surveys, it systematically links architectural design choices to operational enforceability and emerging EHDS governance requirements, revealing that most existing systems remain at a proof-of-concept stage with limited real-world assurance.

## Supplementary material

10.2196/88536 Checklist 1 PRISMA checklist.

## Abbreviations

AI: artificial intelligence
DID: decentralized identifier
DLT: distributed ledger technology
EHDS: European Health Data Space
EHR: electronic health record
EU: European Union
EUDI: European Digital Identity
FHIR: Fast Healthcare Interoperability Resources
GDPR: General Data Protection Regulation
HIE: Health Information Exchange
HL7: Health Level 7
MeSH: Medical Subject Headings
NFT: nonfungible token
PEP: policy enforcement point
PHR: personal health record
PICOS: Population, Intervention, Comparison, Outcome, and Study type
PRISMA: Preferred Reporting Items for Systematic Reviews and Meta-Analyses
QI: quality improvement
TEHDAS 2: Towards the European Health Data Space
VC: verifiable credential
